# Vertebrate Paralogous MEF2 Genes: Origin, Conservation, and Evolution

**DOI:** 10.1371/journal.pone.0017334

**Published:** 2011-03-04

**Authors:** Wenwu Wu, Stefan de Folter, Xia Shen, Wenqian Zhang, Shiheng Tao

**Affiliations:** 1 College of Life Science, Northwest A&F University, Yangling, Shaanxi, China; 2 Bioinformatics Center, Northwest A&F University, Yangling, Shaanxi, China; 3 Laboratorio Nacional de Genómica para la Biodiversidad (Langebio), Centro de Investigación y de Estudios Avanzados del Instituto Politécnico Nacional (CINVESTAV-IPN), Campus Guanajuato, Guanajuato, Mexico; 4 College of Drug Research, Shaanxi University of Chinese Medicine, Xi'an, Shaanxi, China; University of Münster, Germany

## Abstract

**Background:**

The myocyte enhancer factor 2 (MEF2) gene family is broadly expressed during the development and maintenance of muscle cells. Although a great deal has been elucidated concerning MEF2 transcription factors' regulation of specific gene expression in diverse programs and adaptive responses, little is known about the origin and evolution of the four members of the MEF2 gene family in vertebrates.

**Methodology/Principal Findings:**

By phylogenetic analyses, we investigated the origin, conservation, and evolution of the four MEF2 genes. First, among the four MEF2 paralogous branches, MEF2B is clearly distant from the other three branches in vertebrates, mainly because it lacks the HJURP_C (Holliday junction recognition protein C-terminal) region. Second, three duplication events might have occurred to produce the four MEF2 paralogous genes and the latest duplication event occurred near the origin of vertebrates producing MEF2A and MEF2C. Third, the ratio (*K_a_*/*K_s_*) of non-synonymous to synonymous nucleotide substitution rates showed that MEF2B evolves faster than the other three MEF2 proteins despite purifying selection on all of the four MEF2 branches. Moreover, a pair model of M0 versus M3 showed that variable selection exists among MEF2 proteins, and branch-site analysis presented that sites 53 and 64 along the MEF2B branch are under positive selection. Finally, and interestingly, substitution rates showed that type II MADS genes (i.e., MEF2-like genes) evolve as slowly as type I MADS genes (i.e., SRF-like genes) in animals, which is inconsistent with the fact that type II MADS genes evolve much slower than type I MADS genes in plants.

**Conclusion:**

Our findings shed light on the relationship of MEF2A, B, C, and D with functional conservation and evolution in vertebrates. This study provides a rationale for future experimental design to investigate distinct but overlapping regulatory roles of the four MEF2 genes in various tissues.

## Introduction

The myocyte enhancer factor 2 (MEF2) gene family, which belong to the evolutionarily ancient MADS (MCM1, AGAMOUS, DEFICIENS, and SRF)-box superfamily [1]–[4], has four members referred to as MEF2A, B, C, and D located on different chromosomes in vertebrate genomes [5], [6]. Of the four MEF2 members, all can be tissue-specific alternatively spliced, producing multiple isoforms which have significant functional differences [1]–[4]. They recognize and bind to the consensus DNA sequence CTA(A/T)_4_TAG/A as homo- or heterodimers via a 56-amino acid domain (*i.e.* MADS-box) [7], [8]. Adjacent to the MADS-box is a 29-amino acid extension, referred to as the MEF2-specific (MEF2s) domain, which contributes to high-affinity DNA binding and dimerization with other homologous MEF2 proteins and facilitates interactions with other cofactors [9], [10]. The C-terminal of MEF2 proteins, which is subject to complex patterns of alternative splicing, contains the transcriptional activation domain to promote signal transduction and/or regulate target gene transcription [9], [11]–[13].

The four MEF2 proteins display distinct but overlapping expression patterns and regulate the intricate temporal and spatial pattern of gene expression in body development and maintenance [14]–[16]. The well-established roles of MEF2 in muscle development are to control myogenesis and morphogenesis by cooperating with myogenic bHLH factors (*e.g.* MyoD, myogenin) [2], [17], [18], homeobox proteins (*e.g.* tinman, Gax) [19], [20], and/or GATA factors (*e.g.* GATA4) [21], [22]. Other important functions crucially dependent on MEF2 factors have also been elucidated since their discovery. Among these are the regulation of nervous system during both development and injury repair [23], [24], multiple roles in the immune system [25], [26], adipocytes [27], endothelium [28], [29], and chondrocytes and bones [30]–[32]. In the case of MADS proteins in plants, a lot of studies have been focused not only on the functional level, such as revealing ABCDE model for flower organ identity (for review, see [33]–[35]) in Arabidopsis, but also on MADS phylogeny [36]–[39] as well as on natural selection with a particular focus on adaptive evolution [40]–[42].

In contrast to the functions of MEF2 proteins in vertebrates and evolutionary analyses of the MADS family in plants, which have been the subject of extensive research, little is known about the evolutionary relationship of the four MEF2 proteins. At present, we only know that MEF2 proteins share over 65% amino acid identity in the MEF2s domain, and over 90% similarity in the MADS-box in contrast to only about 50% similarity with other MADS factors such as SRF (serum response factor) [2], [38], [43]–[45], which is closely associated with specific DNA binding [46].

Here, we investigate the duplication events and evolutionary rates of the four MEF2 proteins, particularly Darwinian selection on the four MEF2 branches and on the sites in MEF2 sequences and in particular branches. The study strongly improves our understanding of MEF2 conservation and evolution in vertebrates, and the findings may be laid for future experimental dissection of the function of the four MEF2 members.

## Results

### Phylogeny of MEF2 genes

**Figure 1:**
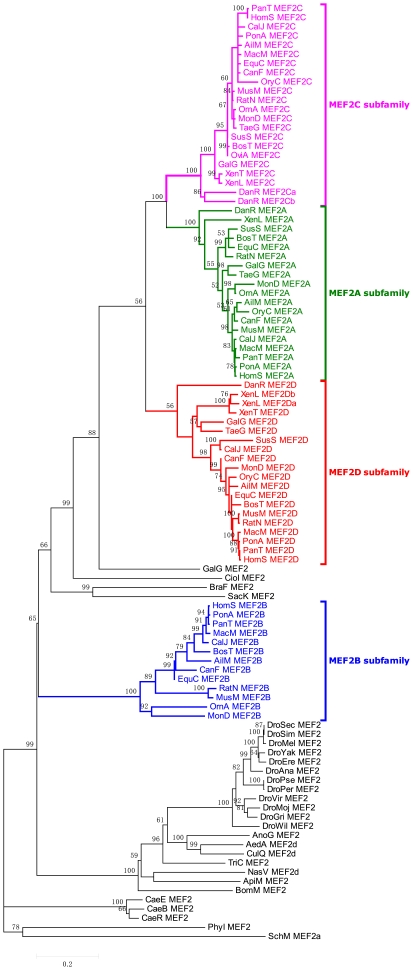
The phylogeny of MEF2 proteins inferred by the neighbor-joining method with Poisson-correction (PC) distance. The scale bar indicates the number of amino acid substitutions per site. The four MEF2 branches in vertebrates are highlighted; bootstrap percentages are indicated on branches supported by a plurality of bootstrap replicates. Leaves are comprised of brief species name and MEF2 type.

The data set of 102 MEF2 protein sequences was aligned to produce the phylogeny (see Figure 1) of MEF2 genes by using the Neighbor-joining (NJ) method [47] (see Materials and methods), and this NJ tree is broadly consistent with the tree constructed by Bayesian method [48] (see Figure S1). The phylogeny shows that MEF2B is the most distant from the other three MEF2 proteins in vertebrates, and MEF2C and MEF2A are more closely tied to each other than to MEF2D and MEF2B genes. In line with MEF2A closely tied to MEF2C, some common types of alternative splicing have been observed for MEF2A and MEF2C transcripts [3], [5], [18]. For example, there have been found 16 and 17 transcripts, respectively, for MEF2A and MEF2C genes in *Pan troglodytes*, and most of them have similar transcriptional splicing patterns.

Interestingly, MEF2C and MEF2D had another independent duplication event in the species *Danio rerio* and *Xenopus laevis*, respectively, producing five paralogous MEF2 proteins in the two species (see Figure 1). According to Sonnhammer's new notion [49] on paralogy and orthology, MEF2Ca and MEF2Cb can be regarded as inparalogs to each other, outparalogs to the other three MEF2 proteins in *Danio rerio*, and co-orthologs to MEF2C protein in other vertebrates, as well as MEF2Da and MEF2Db in *Xenopus laevis*. In contrast to gene duplication, gene loss presumably occurred in some species, such as MEF2B loss in *Oryctolagus cuniculus* (see Figure 1).

### Pairwise estimates of natural selection on MEF2A-D in humans and mice

Nucleotide changes in protein-coding regions of genes are of importance to the conservation and evolution of protein function. Dealing with nucleotide changes, it is necessary to discriminate between changes that affect the amino acid sequence (nonsynonymous substitution) from changes that do not affect amino acid sequence (synonymous substitution). The ratio (*ω = K_a_/K_s_*) of nonsynonymous to synonymous substitution rate is a valid measure of natural selection pressure at the protein level [50], with *ω*<1, *ω*>1, and *ω* = 1 representing purifying selection, positive selection, and neutral evolution, respectively [51].

Synonymous and nonsynonymous substitution rates and their ratios for MEF2A-D protein coding regions are presented in Table 1. All the four *ω* ratios are much lower than one (*ω*<0.2), indicating that MEF2A-D proteins are subject to strong purifying selection to maintain protein function. However, among the four proteins, MEF2B evolves at an even higher rate with much greater nonsynonymous and synonymous substitution rates than the other three MEF2 proteins, and the other three proteins evolve at the same order of magnitude level, though MEF2A has a twice bigger *ω* ratio compared to MEF2C.

**Table 1:** Rates of synonymous (*K_s_*) and nonsynonymous (*K_a_*) nucleotide substitutions (± standard errors) and their ratios (*ω*) for MEF2 protein-coding regions.

Gene	Codons	κ	ω	K_a_	K_s_
MEF2A	489	2.01	0.052	0.0184±0.0042	0.3533±0.0383
MEF2B	347	3.35	0.193	0.1578±0.0157	0.8193±0.1044
MEF2C	465	2.31	0.024	0.0059±0.0024	0.2435±0.0312
MEF2D	606	2.18	0.038	0.0146±0.0036	0.3832±0.0444

Note: All rates are based on comparisons between human and mouse MEF2 coding regions. κ indicates the ratio of the transition to transversion rates.

One of the unresolved issues about the MEF2 family is whether the increased *ω* ratio of MEF2B reflects (i) simply a long-term accumulation under a relaxed selection pressure, or (ii) an abrupt increase in an episodic period for functional divergence following the duplication event. The other question is whether or not some sites in the MEF2 family or in some particular MEF2 branches are under positive selection. In the following, we will focus on variable natural selection among the four MEF2 branches, MEF2 sites, and the sites along particular MEF2 branches to test these scenarios.

### Natural selection among the four MEF2A-D branches

We assumed variable *ω* evolutionary ratios among MEF2 branches in MEF2 phylogeny, and then tested for a significant difference of the ratios based on Likelihood Ratio Test (LRT) (see Materials and methods) [51], [52]. The null hypothesis (H0) is that the evolutionary ratios for the MEF2 family are all simply due to underlying uniform mutation rates (*i.e. ω* is identical across all the branches of the MEF2 phylogeny). Under the H0 model (see Table 2), the estimate of *ω* is 0.012, indicating that the evolution of all the MEF2 members was dominated by strong purifying selection which is consistent with previous results (see Table 1). Given that no significant difference of H1 versus H0 was detected, the increased *ω* rate of MEF2B is likely from a long-term accumulation under a relaxed selection pressure on MEF2B. In addition, among the six alternatives to H0, H5 and H6 for MEF2A and MEF2C branches both with p-value<0.01 suggest that selection pressure (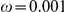) on MEF2A and MEF2C is significantly stronger than on the other two MEF2 branches consistent with previous results (see Table 1).

**Table 2:** Parameter estimates under branch-specific models among the four MEF2 branches for MADS and MEF2s coding regions.

Branch-specific Models	ω_B_	ω_ADC_	ω_D_	ω_AC_	ω_C_	ω_A_		2Δ	*p-value*
H0: ω_B_ = ω_ADC_ = ω_D_ = ω_AC_ = ω_C_ = ω_A_	0.012	0.012	0.012	0.012	0.012	0.012	−1693.33		
H1: ω_B_≠ω_ADC_ = ω_D_ = ω_AC_ = ω_C_ = ω_A_	0.073	0.013	0.013	0.013	0.013	0.013	−1692.44	1.78	0.182
H2: ω_B_ = ω_D_ = ω_AC_ = ω_C_ = ω_A_≠ω_ADC_	0.013	0.073	0.013	0.013	0.013	0.013	−1692.44	1.78	0.182
H3: ω_B_ = ω_ADC_ = ω_AC_ = ω_C_ = ω_A_≠ω_D_	0.012	0.012	0.051	0.012	0.012	0.012	−1692.53	1.61	0.205
H4: ω_B_ = ω_ADC_ = ω_D_ = ω_C_ = ω_A_≠ω_AC_	0.013	0.013	0.013	0.012	0.013	0.013	−1693.43	0.20	0.655
H5: ω_B_ = ω_ADC_ = ω_D_ = ω_AC_ = ω_A_≠ω_C_	0.012	0.012	0.012	0.012	0.001	0.012	−1689.91	6.84	0.0089**
H6: ω_B_ = ω_ADC_ = ω_D_ = ω_AC_ = ω_C_≠ω_A_	0.018	0.018	0.018	0.018	0.018	0.001	−1686.80	13.05	0.0003**

Note: The topology and branch-specific *ω* ratios are presented in Figure S2. The degree of freedom (df) is 1 for the comparisons of null model H0 versus the alternative model from H1 to H6.

**Significance with 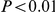.

### Natural selection among MEF2A-D sites

Strong purifying selection dominates the four MEF2 branches regardless of relative relaxed purifying selection on the MEF2B branch, however, whether some sites in MEF2 sequences under adaptive evolution or variable selection are still unknown. To test these, we conducted the following pairs of models from PAML4 [51], [53]: M0 versus M3, M1a versus M2a, and M7 versus M8, and the results are presented in Table 3. For both pair models of M1a versus M2a and M7 versus M8, none of the p-values by LRT are less than 0.01, suggesting that no sites in MEF2 proteins are under positive selection. However, for the pair model of M0 versus M3, the LRT (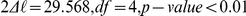) suggests that there are indeed certain sites under highly variable selection pressures across MEF2 proteins. In summary, the analyses show that although none of sites in MEF2 proteins are under positive selection, variable selection pressures exist among MEF2 sites.

**Table 3:** Parameter estimates under site pair models for the MADS and MEF2s coding regions.

Model	ω	Parameter estimates	PSS		2Δ
Model 0(one-ratio)	0.012	ω = 0.077	none	−1693.329	29.568**
Model 3(discrete)	0.024	p: 0.467 0.425 0.109	none	−1678.545	
		ω: 0.001 0.015 0.156			
Model 1a	0.019	p: 0.989 0.011	not allowed	−1689.428	0
(NearlyNeutral)		ω: 0.008 1.000			
Model 2a	0.019	p: 0.989 0.011 0.000	none	−1689.428	
(PositiveSelection)		ω: 0.008 1.000 7.394			
		(note that p[2] is zero)			
Model 7(beta)	0.015	p = 0.160 q = 9.200	not allowed	−1674.351	0
Model 8(beta&ω)	0.015	p0 = 0.999 p = 0.160 q = 9.200	none	−1674.352	
		(p1 = 0.00001) ω = 1.899			
		(note that p1 is nearly zero)			

Note: The *ω* represents for *K_a_/K_s_* that is the average of selection across all sites in the MEF2 coding regions. PSS represents the number of sites under positive selection.

**Significance with 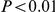.

### Natural selection among sites along particular MEF2A, B, C, and D branches

Given that positive selection often operates only on a few amino acid sites along particular branches [53], we employed branch-site specific Model A (see Materials and methods) to detect whether some sites along particular MEF2 branches are under positive selection, and the results are presented in Table 4. Along MEF2A and MEF2C branches, there are no sites with *ω* ratio greater than 1 demonstrating that none of the sites in the branches underwent adaptive evolution. However, along MEF2B and MEF2D branches, both of the LRTs are significant less than 0.05, demonstrating that some sites along the MEF2B and MEF2D branches underwent adaptive evolution. The sites are 53 and 64 under positive selection with the posterior probability >95% along the MEF2B branch as well as 50 along the MEF2D branch (see Discussion).

**Table 4:** Parameter estimates under branch-site models along particular MEF2 branch.

Branch-site models		Parameter estimates	PSS		2Δ
Foreground MEF2A Branch	Model A H0	ω_0_ = 0.008 P_0_ = 0.989 ω_1_ = 1.000 P_1_ = 0.011	Not allowed	−1689.428	0
	(ω_2_ = 1)	ω_2a fore_ = 1.000 ω_2a back_ = 0.008 P_2a_ = 0.000			
		ω_2b fore_ = 1.000 ω_2b back_ = 1.000 P_2b_ = 0.000			
	Model A H1	ω_0_ = 0.008 P_0_ = 0.989 ω_1_ = 1.000 P_1_ = 0.011	None	−1689.428	
		ω_2a fore_ = 1.000 ω_2a back_ = 0.008 P_2a_ = 0.000			
		ω_2b fore_ = 1.000 ω_2b back_ = 1.000 P_2b_ = 0.000			
Foreground MEF2C Branch	Model A H0	ω_0_ = 0.008 P_0_ = 0.989 ω_1_ = 1.000 P_1_ = 0.011	Not allowed	−1689.428	0
	(ω_2_ = 1)	ω_2a fore_ = 1.000 ω_2a back_ = 0.008 P_2a_ = 0.000			
		ω_2b fore_ = 1.000 ω_2b back_ = 1.000 P_2b_ = 0.000			
	Model A H1	ω_0_ = 0.008 P_0_ = 0.989 ω_1_ = 1.000 P_1_ = 0.011	None	−1689.428	
		ω_2a fore_ = 1.000 ω_2a back_ = 0.008 P_2a_ = 0.000			
		ω_2b fore_ = 1.000 ω_2b back_ = 1.000 P_2b_ = 0.000			
Foreground MEF2B Branch	Model A H0	ω_0_ = 0.011 P_0_ = 0.832 ω_1_ = 1.000 P_1_ = 0.011	Not allowed	−1686.781	3.062*
	(ω_2_ = 1)	ω_2a fore_ = 1.000 ω_2a back_ = 0.011 P_2a_ = 0.155			
		ω_2b fore_ = 1.000 ω_2b back_ = 1.000 P_2b_ = 0.000			
	Model A H1	ω_0_ = 0.011 P_0_ = 0.871 ω_1_ = 1.000 P_1_ = 0.011	9 (0.851) 12 (0.862)	−1685.250	
		ω_2a fore_ = 8.299 ω_2a back_ = 0.011 P_2a_ = 0.155	14(0.795) 51(0.760)		
		ω_2b fore_ = 8.299 ω_2b back_ = 1.000 P_2b_ = 0.002	53(0.996**) 64(0.964*)		
			73(0.647) 85(0.614)		
			90(0.694)		
Foreground MEF2D Branch	Model A H0	ω_0_ = 0.007 P_0_ = 0.920 ω_1_ = 1.000 P_1_ = 0.011	Not allowed	−1682.317	3.360*
	(ω_2_ = 1)	ω_2a fore_ = 1.000 ω_2a back_ = 0.007 P_2a_ = 0.068			
		ω_2b fore_ = 1.000 ω_2b back_ = 1.000 P_2b_ = 0.001			
	Model A H1	ω_0_ = 0.011 P_0_ = 0.944 ω_1_ = 1.000 P_1_ = 0.011	9(0.909) 50(0.963*)	−1680.637	
		ω_2a fore_ = 999.000 ω_2a back_ = 0.011 P_2a_ = 0.045			
		ω_2b fore_ = 999.000 ω_2b back_ = 1.000 P_2b_ = 0.001			

Note: Model A H0 is specified using fixed *ω*_2_ = 1. The p-value of Model A H0 versus Model A H1 for the MEF2B and MEF2D branches is 0.040 and 0.033, respectively, which are considered to be statistically significant.

### Evolution of type I and type II MADS factors

**Figure 2:**
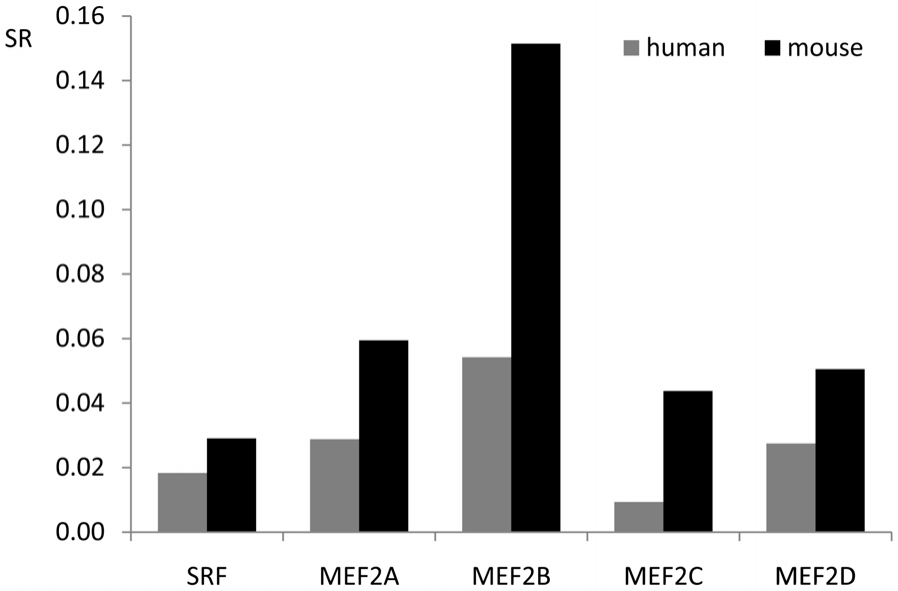
Substitution rates of SRF and MEF2A-D coding regions in the human and mouse genomes. SR on Y-axis represents for substitution rate, that is, mutation rate per site across the corresponding coding region. Dog and cow are used as outgroups to identify substitution sites.

There are two types of MADS factors in plants and animals, called type I (SRF-like) and type II (MEF2-like) MADS factors [54]. To our knowledge, type I MADS factors evolve faster than type II MADS factors in plants [55], [56]. However, little is known about the evolutionary rate of the two types in animals. Substitution rates of SRF and MEF2A-D genes in the human and mouse genomes are presented in Figure 2. From the figure, mSRF (referred to as SRF in mice) evolves faster than its corresponding orthologous hSRF (referred to as SRF in humans). Likewise, the evolutionary rate of the MEF2 family also reveals the same pattern that MADS factors evolve faster in mice than in humans. When comparing paralogous genes, the substitution rate of MEF2B is much higher than that of SRF, MEF2A, MEF2C, and MEF2D, demonstrating that MEF2B evolves faster than SRF as well as MEF2A, MEF2C, and MEF2D. In support of this result, the analysis of indels revealed that MEF2B bears 7 short fragment deletions and 1 fragment insertion, which is more than the other MADS-box factors bearing in the mouse genome. Furthermore, there are also slight differences in evolutionary rate among MEF2A, MEF2C, MEF2D, and SRF between mice and humans.

## Discussion

The four members of the MEF2 gene family are broadly expressed in different but overlapping patterns during embryogenesis and postnatal development as well as throughout adulthood in vertebrates [2], [15], [16]. Here, we analyzed the evolutionary relationship of the four MEF2 proteins. Phylogenetic analysis shows that MEF2B is the most distant from the other three MEF2 proteins in vertebrates, and MEF2A and MEF2C originated from the latest duplication event near the origin of vertebrates. Lineage-specific analysis of the MEF2 gene family shows that a long-term accumulation of substitutions after the duplication led to the MEF2B branch evolving faster than the other MEF2 branches. In addition, site-specific analysis of the MEF2 gene family shows that although all the sites in MEF2 proteins are clearly constrained by purifying selection, variable purifying selection appears in the MADS and MEF2s regions of MEF2 proteins. In contrast to strong purifying selection, branch-site analysis shows that sites 53 and 64 along the MEF2B branch and 50 along the MEF2D branch are under positive selection. Furthermore, analysis of substitution rates for SRF and MEF2A-D shows that SRF evolves as slowly as MEF2 proteins except for MEF2B.

### Duplication of MEF2 genes

**Figure 3:**
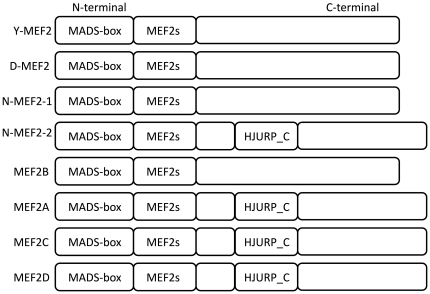
Domain regions in MEF2 proteins. N-terminal and C-terminal are marked on the left and right, respectively. Y-MEF2, D-MEF2, and N-MEF2-1 and N-MEF2-2 represent for MEF2 proteins of yeast, Drosophila, and Nematostella, respectively, in invertebrates. MEF2A-D represents for MEF2 proteins in vertebrates.

MEF2B is the most distant among the four MEF2 members in vertebrates, which is mainly because of lacking the HJURP_C region (see Figure 3), but also other sequential characters. In support of this, the MEF2A-D phylogenetic tree (see Figure S2) constructed by the alignment of only the MADS and MEF2s regions also proves that MEF2B is the most distant. In addition, an invertebrate animal called *Nematostella vectensis* has two MEF2-type genes (see Figure 3) [57]: one has no HJURP_C region similar to MEF2B in vertebrates; and the other has the HJURP_C region similar to MEF2A, C, and D. To our knowledge, the origin of the HJURP_C region is far much later than MADS/MEF2s domains because that the HJURP was just found in higher eukaryotes [58]. Given that original MEF2 proteins have no such HJURP_C region, we presume that the origin of MEF2B is more ancient than the other three MEF2 proteins which include the HJURP_C region, and the three MEF2 proteins should share a common ancestor also including the HJURP_C region. According to the presence of two MEF2-type genes in the invertebrate species *Nematostella vectensis*: one has the HJURP_C region and the other does not, we further presume that the first duplication event occurred before the origin of vertebrates producing two copies of MEF2 genes, and in the following evolutionary process, one finally became extant MEF2B, the other was inserted by the HJURP_C region which lies at C-terminal to the MADS/MEF2s regions and this MEF2 gene might be the most recent common ancestor of MEF2D, A, and C. Thereafter, such MEF2 gene had two duplication events to produce MEF2D, A, and C near the origin of vertebrates.

In relation to gene duplication patterns, MEF2A-D genes seem to originate from interchromosomal duplications considering that the four MEF2 genes are distributed on different chromosomes [5], [6].

### Functional constraints on MEF2A-D

The pairwise approach proposed by Yang et al. [59] is an efficient iterative means for computing synonymous and nonsynonymous substitution rates. By this approach, we found that, in addition to MEF2A-D under purifying selection, MEF2B evolves faster than the other three MEF2 proteins. A reasonable explanation is that the functional constraint on MEF2B is lower than on the other three MEF2 genes. This could be why few mutant MEF2B phenotypes have ever been reported. In contrast, many mutant phenotypes have been known for MEF2A, C, and D from mice [60]. For example, targeted inactivation of MEF2A, C, and D in mice results in cardiac lethality [61], embryonic lethality [62], and a failure of normal bone development [30], respectively. Poor mutant phenotypes for MEF2B gene could be because of its possible functional redundancy with other MEF2 genes and thus MEF2B probably functions as a potential candidate for the other MEF2 proteins. In support of this hypothesis, the alternative splicing of MEF2B transcripts is altered in MEF2C mutant embryos [63], and a significant upregulation of MEF2B expression was observed [62]. However, double or multiple knockouts of MEF2B and other MEF2 genes, such as inactivation of MEF2C and MEF2B in embryos, will be especially interesting and would provide more information on the roles of MEF2B.

Although all the MEF2 genes are subject to purifying selection, different sites of MEF2 genes including MADS and MEF2s regions are under variable purifying selection. Site model analysis shows that none common sites in the four MEF2 branches are under positive selection, whereas branch-site analysis shows that sites 53 and 64 along MEF2B branch and 50 along MEF2D branch are under positive selection. Of interest, at both sites 53 and 64, amino acid R is present in MEF2B branch in contrast to K in the other MEF2 branches. Elegant studies from Alvarez-Buylla's group [41], [42] presented that some positions in the K domain of MIKC proteins in plants, which has similar functions (e.g. dimerization) as MEF2s region in MEF2 proteins, are under positive selection involved in both dimerization and α-β folding; whereas functional differences between R and K on the two sites (i.e. 53 and 64) are little known. In the case of site 50 along the MEF2D branch, residue H is present in contrast to residue S in the homologous site along the other MEF2 branches. However, the *ω* ratio for this site is 999 because of the rate of synonymous substitution *K_s_*  =  0 and thus *K_a_/K_s_* is represented as 999.

### Faster evolutionary rate of MADS factors in mice than in humans

MADS factors evolve faster in mice than in humans. One reasonable explanation is that mice with a shorter generation length than humans would undergo more germ-line cell divisions and thus accumulate a larger number of mutations in unit time, which would lead to a larger number of substitutions in mice than in humans [64], [65]. However, a shorter generation length of about 80 times in mice than in humans [65] is largely inconsistent with only 1–3 times the substitution rates of MADS factors in mice than in humans. Therefore, there should be other ways to affect the accumulation of substitutions, such as mutation repair efficiency [66], rate of cell division [67], and weight-specific metabolic rate [68]. Furthermore, essential functions of MADS factors whether in mice or humans, usually do not suffer deleterious mutations in MADS factors and thus natural selection would eliminate such mutations. Given these causes, MADS factors evolve just 1–3 times faster in mice than in humans. However, which one or more causes play pivotal roles in constraining the evolutionary rate will need to be evaluated with further research.

### Similar functional conservation between type I and type II MADS factors in animals

Based on evolutionary analysis of MADS-box genes in plants, two groups [55], [56] concluded that type I MADS genes evolve much faster than type II MADS genes. Our findings in animals, however, indicate that type I MADS genes, evolve as slowly as type II MADS genes. Unlike, possibly, less functional importance or functional redundancy of type I MADS factors in plants [69], type I MADS factor usually represented as only one SRF-like MADS factor in animals is expressed ubiquitous and plays essential roles in cell differentiation and growth [70]–[72]. For example, SRF-null mice die before gastrulation and do not form mesoderm [73], demonstrating that SRF is an obligatory transcription factor and thus mutation of SRF would lead to injury, illness, and even death of the organism. In contrast to SRF, the four members of the MEF2 family are mainly involved in tissue-restricted gene expression of three muscle cells but also of other cells, including T-lymphocytes, B-cells, chondrocytes, and neural crest cells [26], [30], [31], [74]–[77], and they also play essential roles in gene regulation. These considerations explain quite well why SRF evolves at nearly the same conservational level with MEF2A, MEF2C, and MEF2D, except slower than MEF2B.

In summary, we have constructed the phylogeny of the MEF2 genes, and revealed that the function of MEF2B is somewhat less important than the other three MEF2 members in vertebrates, which is consistent with functional research from previous experimental observations. To circumvent putative problems with redundancy between MEF2B and other MEF2 proteins, generation of double or multiple MEF2 knockouts is especially interesting and would provide a deeper comprehension of the different and/or overlapping functional roles of the four MEF2 members in vertebrates.

## Materials and Methods

### Data collection and alignment

Orthologous and paralogous MEF2 sequences (As a total of 102 sequences, see File S1) were obtained from The National Center for Biotechnology Information (NCBI) using BLASTP, TBLASTN, and key words searches [74]. The MEF2 amino acid sequences were aligned by the program MUSCLE, and poorly aligned positions and divergent regions (*e.g.* a number of indels and/or mismatches) were eliminated by the program Gblocks in combination with manual edition. The alignment result (see File S2) was used to construct the MEF2 phylogeny. On the other hand, a Perl script was written to capture the open reading frames (excluding 5′-UTR and 3′-UTR) by using the corresponding MEF2 protein sequences against the corresponding mRNA sequences (see File S3). Thereafter, MEF2 coding sequences were aligned according to the previous alignment of MEF2 protein sequences by ClustalW as implemented in MEGA4 [78]. Because the regions C-terminal to MEF2s domains are two divergent among the four MEF2A-D branches, it is not appropriate to calculate nonsynonymous and synonymous substitution rates and their ratios (*ω = K_a_/K_s_*) [79], therefore, just the MADS and MEF2s domains were used for the analyses. The alignment result (see File S4) of MADS and MEF2s coding regions was used to calculate *K_a_*, *K_s_*, and their ratios by PAML4 (Phylogenetic Analysis by Maximum Likelihood, version 4).

### Phylogenetic analysis

The MEF2 phylogenetic tree was constructed by Neighbor-Joining (NJ) method with 500 bootstrap replicates, poisson-correction (PC) distance, and pairwise deletion options as implemented in MEGA 4 [78]. In addition, MrBayes 3.1 [48] with default model and priors was used to construct MEF2 Bayesian phylogenetic tree. Searches were started from a random tree (nruns = 1) with 4 heated chains (temp = 0.05) and 300,000 iterations, the initial 5,000 trees were discarded, and finally a consensus tree using the Bayesian posterior probabilities (PPs) to evaluate branch support was constructed. The consensus Bayesian tree (see Figure S1) is broadly consistent with the former NJ tree.

### Detection of evolutionary rates for MADS and MEF2s coding regions

To test whether there were different evolutionary rates among MEF2A-D proteins in vertebrates, the YN00 program [59] of PAML4 [53] was used to estimate substitution rates of MEF2 coding sequences by pairwise calculation of *K_a_/K_s_* between mice and humans (see Table 1). To our knowledge, a high evolutionary rate is thought to originate from two possible ways: one is simply a long-term accumulation of substitutions because of relaxed natural selection; the other is an abrupt increase of substitutions in an episodic period because of functional divergence. To test which scenario brings the increase of MEF2B evolutionary rate, the CODEML program of PAML4 [53] was used to implement models that allow for different *ω* parameters in different parts of the MEF2A-D phylogeny (see Figure S2). The simplest model, referred to as null hypothesis H0, assumes the same *ω* ratio for all branches in the phylogeny. Other models, referred to as alternatives, specify independent *ω* ratio for the corresponding branch in the phylogeny (see Table 2). The likelihood ratio test (LRT) [52] was applied to measure the statistical significance of each pair of nested models.

Since positive selection is likely to act on a small subset of sites in a protein and thus averages of substitution rates across a protein with lower than 1 may not represent that all the sites in the protein are under negative selection. Besides, even though all the sites in a protein are under negative selection, various negative selection pressures still may appear in different domains in a protein. To test whether some sites in MADS and MEF2s regions are under positive selection, we used two pair models from the CODEML program [53]: M1a (Nearly Neutral) against M2a (Positive Selection); and M7 (beta) against M8 (beta & *ω*). M1a allows two classes of *ω* sites: negative sites with *ω*_0_<1 estimated from our data and neutral sites with *ω*_1_ = 1, whereas M2a adds a third class with *ω*_2_ possibly >1 estimated from our data. M7 allows ten classes of *ω* sites between 0 and 1 according to a beta distribution with parameters *p* and *q*, whereas M8 adds an additional class with *ω* possibly >1 as M2a does. In both comparisons, degree of freedom (*df*) is 2. In addition, to test whether variable selection pressures exist among MADS/MEF2s sites, we used a pair model also from the CODEML program [53]: M0 (one ratio) against M3 (discrete). M0 specifies a single *ω* ratio for all MEF2 coding sites, whereas M3 specifies MEF2 coding sites into 3 discrete classes. Degree of freedom for this comparison is 4.

In addition, to reveal whether there are some sites along particular MEF2 branches, we also did branch-site analyses employing the Test 2 [80] of the null model A H0 (model  =  2 NSsites  =  2) with *ω*_2_ fixed to 1 in comparison to alternative model A H1 with *ω*_2_ to be estimated [53]. In contrast to 3.84 for 5% and 6.63 for 1% for 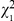, the critical values are 2.71 at 5% and 5.41 at 1% [53] given that the null distribution (the branch-site model we used here) is the 50∶50 mixture of point mass 0 and 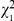.

To compare evolutionary rate between type I and type II MADS factors in animals, substitution rates (see Figure 2) of SRF and MEF2A-D were calculated in humans and mice by using dogs and cows as outgroups. Here, substitution rate was simply determined as mutation rate per site [81] across coding sequences.

## Supporting Information

File S1 A total of 102 MEF2 protein sequences.
(FASTA)

File S2 Alignment result for the 102 MEF2 protein sequences.
(PDF)

File S3 A total of 102 MEF2 coding sequences excluding 5′-UTR and 3′-UTR.
(FASTA)

File S4 Alignment result of the MADS and MEF2s coding regions for the MEF2A, B, C, and D branches.
(PDF)

Figure S1 MEF2 Bayesian tree.
(PDF)

Figure S2 D*ifferent ω* parameters for different parts of the MEF2A-D phylogeny.
(PDF)
