# Supplementary material for: Vertebrate Paralogous MEF2 Genes: Origin, Conservation, and Evolution
Source: PLoS One. 2011 Mar 4;6(3):e17334. doi: 10.1371/journal.pone.0017334 (PMC3048864; doi:10.1371/journal.pone.0017334)

```
                      .   *** *** *::*** *** ****:****:*   ::*:**:*:.:*::**:***:.**:*:      :      .
CanF_MEF2A   MGRKKIQIQRIMDERNRQVTFTKRKFGLMKKAYELSVLCDCEIALIIFNSSNKLFQYASTDMDKVLLKYTEYNEPHESRTNSDIVETLRKKGLNGCESPD   100
MacM_MEF2A   MGRKKIQITRIMDERNRQVTFTKRKFGLMKKAYELSVLCDCEIALIIFNSSNKLFQYASTDMDKVLLKYTEYNEPHESRTNSDIVETLRKKGLNGCESPD   100
PanT_MEF2A   MGRKKIQITRIMDERNRQVTFTKRKFGLMKKAYELSVLCDCEIALIIFNSSNKLFQYASTDMDKVLLKYTEYNEPHESRTNSDIVETLRKKGLNGCESPD   100
SusS_MEF2A   MGRKKIQITRIMDERNRQVTFTKRKFGLMKKAYELSVLCDCEIALIIFNSSNKLFQYASTDMDKVLLKYTEYNEPHESGTNSDIVEALNKKEHRGCDSPD   100
EquC_MEF2A   MGRKKIQITRIMDERNRQVTFTKRKFGLMKKAYELSVLCDCEIALIIFNSSNKLFQYASTDMDKVLLKYTEYNEPHESRTNSDIVEALNKKEHRGCDSPD   100
PonA_MEF2A   MGRKKIQITRIMDERNRQVTFTKRKFGLMKKAYELSVLCDCEIALIIFNSSNKLFQYASTDMDKVLLKYTEYNEPHESRTNSDIVETLRKKGLNGCESPD   100
HomS_MEF2A   MGRKKIQITRIMDERNRQVTFTKRKFGLMKKAYELSVLCDCEIALIIFNSSNKLFQYASTDMDKVLLKYTEYNEPHESRTNSDIVETLRKKGLNGCESPD   100
CalJ_MEF2A   MGRKKIQITRIMDERNRQVTFTKRKFGLMKKAYELSVLCDCEIALIIFNSSNKLFQYASTDMDKVLLKYTEYNEPHESRTNSDIVETLRKKGLNGCESPD   100
AilM_MEF2A   VNTVKIPETRVTEAQAHRVTFTKRKFGLMKKAYELSVLCDCEIALIIFNSSNKLFQYASTDMDKVLLKYTEYNEPHESRTNSDIVETLRKKGLNGCESPD   100
MusM_MEF2A   MGRKKIQITRIMDERNRQVTFTKRKFGLMKKAYELSVLCDCEIALIIFNSSNKLFQYASTDMDKVLLKYTEYNEPHESRTNSDIVETLRKKGLNGCESPD   100
DanR_MEF2A   MGRKKIQITRIMDERNRQVTFTKRKFGLMKKAYELSVLCDCEIALIIFNSSNKLFQYASTDMDKVLLKYTEYNEPHESRTNSDIVEKLRNKGHNDCPSPD   100
GalG_MEF2A   MGRKKIQITRIMDERNRQVTFTKRKFGLMKKAYELSVLCDCEIALIIFNSSNKLFQYASTDMDKVLLKYTEYNEPHESRTNSDIVETLRKKGLNGCESPD   100
RatN_MEF2A   MGRKKIQITRIMDERNRQVTFTKRKFGLMKKAYELSVLCDCEIALIIFNSSNKLFQYASTDMDKVLLKYTEYNEPHESRTNSDIVEALNKKEHRGCDSPD   100
BosT_MEF2A   MGRKKIQITRIMDERNRQVTFTKRKFGLMKKAYELSVLCDCEIALIIFNSSNKLFQYASTDMDKVLLKYTEYNEPHESRTNSDIVEALNKKEHRGCDSPD   100
MonD_MEF2A   MERSDKLVSRKMGER-REVTFTKRKFGLMKKAYELSVLCDCEIALIIFNSSNKLFQYASTDMDKVLLKYTEYNEPHESRTNSDIVETLRKKGLNGCESPD    99
XenL_MEF2A   MGRRKIQITRIMDDRNKQVTFTKRKFGLMKKAYELSVLCDCEIALIIFNSSNKLFQYASTDMDKVLLKYTEYNEPHESRTNSDIVEALNKKENRGCDSPD   100
OrnA_MEF2A   MGRKKIQITRIMDERNRQVTFTKRKFGLMKKAYELSVLCDCEIALIIFNSSNKLFQYASTDMDKVLLKYTEYNEPHESRTNSDIVETLRKKGLNGCESPD   100
TaeG_MEF2A   MGRKKIQITRIMDERNRQVTFTKRKFGLMKKAYELSVLCDCEIALIIFNSSNKLFQYASTDMDKVLLKYTEYNEPHESRTNSDIVETLRKKGLNGCESPD   100
OryC_MEF2A   -----------MDERNRQVTFTKRKFGLMKKAYELSVLCDCEIALIIFNSSNKLFQYASTDMDKVLLKYTEYNEPHESRTNSDIVETLRKKGLNGCESPD    89
CanF_MEF2C   MGRKKIQITRIMDERNRQVTFTKRKFGLMKKAYELSVLCDCEIALIIFNSTNKLFQYASTDMDKVLLKYTEYNEPHESRTNSDIVETLRKKGLNGCDSPD   100
MacM_MEF2C   MGRKKIQITRIMDERNRQVTFTKRKFGLMKKAYELSVLCDCEIALIIFNSTNKLFQYASTDMDKVLLKYTEYNEPHESRTNSDIVETLRKKGLNGCDSPD   100
PanT_MEF2C   MGRKKIQITRIMDERNRQVTFTKRKFGLMKKAYELSVLCDCEIALIIFNSTNKLFQYASTDMDKVLLKYTEYNEPHESRTNSDIVEALNKKENKGCESPD   100
SusS_MEF2C   MGRKKIQITRIMDERNRQVTFTKRKFGLMKKAYELSVLCDCEIALIIFNSTNKLFQYASTDMDKVLLKYTEYNEPHESRTNSDIVETLRKKGLNGCDSPD   100
EquC_MEF2C   MGRKKIQITRIMDERNRQVTFTKRKFGLMKKAYELSVLCDCEIALIIFNSTNKLFQYASTDMDKVLLKYTEYNEPHESRTNSDIVETLRKKGLNGCDSPD   100
PonA_MEF2C   MGRKKIQITRIMDERNRQVTFTKRKFGLMKKAYELSVLCDCEIALIIFNSTNKLFQYASTDMDKVLLKYTEYNEPHESRTNSDIVETLRKKGLNGCDSPD   100
HomS_MEF2C   MGRKKIQITRIMDERNRQVTFTKRKFGLMKKAYELSVLCDCEIALIIFNSTNKLFQYASTDMDKVLLKYTEYNEPHESRTNSDIVETLNKKENKGCESPD   100
CalJ_MEF2C   MGRKKIQITRIMDERNRQVTFTKRKFGLMKKAYELSVLCDCEIALIIFNSTNKLFQYASTDMDKVLLKYTEYNEPHESRTNSDIVETLRKKGLNGCDSPD   100
AilM_MEF2C   MGRKKIQITRIMDERNRQVTFTKRKFGLMKKAYELSVLCDCEIALIIFNSTNKLFQYASTDMDKVLLKYTEYNEPHESRTNSDIVETLRKKGLNGCDSPD   100
MusM_MEF2C   MGRKKIQITRIMDERNRQVTFTKRKFGLMKKAYELSVLCDCEIALIIFNSTNKLFQYASTDMDKVLLKYTEYNEPHESRTNSDIVETLRKKGLNGCDSPD   100
GalG_MEF2C   MGRKKIQITRIMDERNRQVTFTKRKFGLMKKAYELSVLCDCEIALIIFNSTNKLFQYASTDMDKVLLKYTEYNEPHESRTNSDIVETLRKKGLNGCDSPD   100
RatN_MEF2C   MGRKKIQITRIMDERNRQVTFTKRKFGLMKKAYELSVLCDCEIALIIFNSTNKLFQYASTDMDKVLLKYTEYNEPHESRTNSDIVETLRKKGLNGCDSPD   100
BosT_MEF2C   MGRKKIQITRIMDERNRQVTFTKRKFGLMKKAYELSVLCDCEIALIIFNSTNKLFQYASTDMDKVLLKYTEYNEPHESRTNSDIVETLRKKGLNGCDSPD   100
XenT_MEF2C   MGRKKIQITRIMDERNRQVTFTKRKFGLMKKAYELSVLCDCEIALIIFNSTNKLFQYASTDMDKVLLKYTEYNEPHESRTNSDIVETLRKKGLNGCDSPD   100
MonD_MEF2C   MGRKKIQITRIMDERNRQVTFTKRKFGLMKKAYELSVLCDCEIALIIFNSTNKLFQYASTDMDKVLLKYTEYNEPHESRTNSDIVETLRKKGLNGCDSPD   100
XenL_MEF2C   MGRKKIQITRIMDERNRQVTFTKRKFGLMKKAYELSVLCDCEIALIIFNSTNKLFQYASTDMDKVLLKYTEYNEPHESRTNSDIVETLRKKGLNGCDSPD   100
OrnA_MEF2C   MGRKKIQITRIMDERNRQVTFTKRKFGLMKKAYELSVLCDCEIALIIFNSTNKLFQYASTDMDKVLLKYTEYNEPHESRTNSDIVETLRKKGLNGCDSPD   100
TaeG_MEF2C   MGRKKIQITRIMDERNRQVTFTKRKFGLMKKAYELSVLCDCEIALIIFNSTNKLFQYASTDMDKVLLKYTEYNEPHESRTNSDIVETLRKKGLNGCDSPD   100
OviA_MEF2C   MGRKKIQITRIMDERNRQVTFTKRKFGLMKKAYELSVLCDCEIALIIFNSTNKLFQYASTDMDKVLLKYTEYNEPHESRTNSDIVETLRKKGLNGCDSPD   100
OryC_MEF2C   FGYTKVEEHRKVNTNSLMVTFTKRKFGLMKKAYELSVLCDCEIALIIFNSTNKLFQYASTDMDKVLLKYTEYNEPHESRTNSDIVETLRKKGLNGCDSPD   100
DanR_MEF2Ca  MGRKKIQIARIMDERNRQVTFTKRKFGLMKKAYELSVLCDCEIALIIFNSTNKLFQYASTDMDKVLLKYTEYNEPHESRTNSDIVETLRKKGLNGCDSPD   100
DanR_MEF2Cb  MGRKKIQITRIMDERNRQVTFTKRKFGLMKKAYELSVLCDCEIALIIFNSTNKLFQYASTDMDKVLLKYTEYNEPHESRTNSDIVETLRKKGLNGCDSPD   100
CanF_MEF2D   MGRKKIQIQRITDERNRQVTFTKRKFGLMKKAYELSVLCDCEIALIIFNHSNKLFQYASTDMDKVLLKYTEYNEPHESRTNADIIETLRKKGFNGCDSPE   100
MacM_MEF2D   MGRKKIQIQRITDERNRQVTFTKRKFGLMKKAYELSVLCDCEIALIIFNHSNKLFQYASTDMDKVLLKYTEYNEPHESRTNADIIETLRKKGFNGCDSPE   100
PanT_MEF2D   MGRKKIQIQRITDERNRQVTFTKRKFGLMKKAYELSVLCDCEIALIIFNHSNKLFQYASTDMDKVLLKYTEYNEPHESRTNADIIETLRKKGFNGCDSPE   100
SusS_MEF2D   MGRKKIQIQRITDERNRQVTFTKRKFGLMKKAYELSVLCDCEIALIIFNHSNKLFQYASTDMDKVLLKYTEYNEPHESRTNADIIETLRKKGFNGCDSPE   100
EquC_MEF2D   MGRKKIQIQRITDERNRQVTFTKRKFGLMKKAYELSVLCDCEIALIIFNHSNKLFQYASTDMDKVLLKYTEYNEPHESRTNADIIETLRKKGFNGCDSPE   100
PonA_MEF2D   MGRKKIQIQRITDERNRQVTFTKRKFGLMKKAYELSVLCDCEIALIIFNHSNKLFQYASTDMDKVLLKYTEYNEPHESRTNADIIETLRKKGFNGCDSPE   100
HomS_MEF2D   MGRKKIQIQRITDERNRQVTFTKRKFGLMKKAYELSVLCDCEIALIIFNHSNKLFQYASTDMDKVLLKYTEYNEPHESRTNADIIETLRKKGFNGCDSPE   100
CalJ_MEF2D   MGRKKIQIQRITDERNRQVTFTKRKFGLMKKAYELSVLCDCEIALIIFNHSNKLFQYASTDMDKVLLKYTEYNEPHESRTNADIIETLRKKGFNGCDSPE   100
AilM_MEF2D   MGRKKIQIQRITDERNRQVTFTKRKFGLMKKAYELSVLCDCEIALIIFNHSNKLFQYASTDMDKVLLKYTEYNEPHESRTNADIIETLRKK-----DSPE    95
MusM_MEF2D   MGRKKIQIQRITDERNRQVTFTKRKFGLMKKAYELSVLCDCEIALIIFNHSNKLFQYASTDMDKVLLKYTEYNEPHESRTNADIIETLRKKGFNGCDSPE   100
DanR_MEF2D   MGRKKIQIQRITDERNRQVTFTKRKFGLMKKAYELSVLCDCEIALIIFNHSNKLFQYASTDMDKVLLKYTEYNEPHESRTNADIIEALNKKEHRDSESPD   100
GalG_MEF2D   WGAKLLSMLGSGDIQAAAVTFTKRKFGLMKKAYELSVLCDCEIALIIFNHSNKLFQYASTDMDKVLLKYTEYNEPHESRTNADIIETLRKKGFNGCDSPE   100
RatN_MEF2D   MGRKKIQIQRITDERNRQVTFTKRKFGLMKKAYELSVLCDCEIALIIFNHSNKLFQYASTDMDKVLLKYTEYNEPHESRTNADIIETLRKKGFNGCDSPE   100
BosT_MEF2D   MGRKKIQIQRITDERNRQVTFTKRKFGLMKKAYELSVLCDCEIALIIFNHSNKLFQYASTDMDKVLLKYTEYNEPHESRTNADIIETLRKKGFNGCDSPE   100
XenT_MEF2D   MGRKKIQIQRITDERNRQVTFTKRKFGLMKKAYELSVLCDCEIALIIFNHSNKLFQYASTDMDKVLLKYTEYNEPHESRTNADIIETLRKKGFNGCDSPE   100
MonD_MEF2D   MGRKKIQIQRITDERNRQVTFTKRKFGLMKKAYELSVLCDCEIALIIFNHSNKLFQYASTDMDKVLLKYTEYNEPHESRTNADIIETLRKKGFNGCDSPE   100
TaeG_MEF2D   MGRKKIQIQRITDERNRQVTFTKRKFGLMKKAYELSVLCDCEIALIIFNHSNKLFQYASTDMDKVLLKYTEYNEPHESRTNADIIETLRKKGFNGCDSPE   100
OryC_MEF2D   MGRKKIQIQRITDERNRQVTFTKRKFGLMKKAYELSVLCDCEIALIIFNHSNKLFQYASTDMDKVLLKYTEYNEPHESRTNADIIETLRKKGFNGCDSPE   100
XenL_MEF2Db  MGRKKIQIQRITDERNRQVTFTKRKFGLMKKAYELSVLCDCEIALIIFNHSNKLFQYASTDMDKVLLKYTEYNEPHESRTNADIIETLRKKGFNGCDSPE   100
XenL_MEF2Da  MGRKKIQIQRITDERNRQVTFTKRKFGLMKKAYELSVLCDCEIALIIFNHSNKLFQYASTDMDKVLLKYTEYNEPHESRTNADIIETLRKKGFNGCDSPE   100
CanF_MEF2B   MGRKKIQISRILDQRNRQVTFTKRKFGLMKKAYELSVLCDCEIALIIFNSANRLFQYASTDMDRVLLKYTEYSEPHESRTNTDILETLKRRGVGLDGPE-    99
MacM_MEF2B   MGRKKIQISRILDQRNRQVTFTKRKFGLMKKAYELSVLCDCEIALIIFNSANRLFQYASTDMDRVLLKYTEYSEPHESRTNTDILETLKRRGIGLDGPE-    99
PanT_MEF2B   MGRKKIQISRILDQRNRQVTFTKRKFGLMKKAYELSVLCDCEIALIIFNSANRLFQYASTDMDRVLLKYTEYSEPHESRTNTDILETLKRRGIGLDGPE-    99
EquC_MEF2B   MGRKKIQISRILDQRNRQVTFTKRKFGLMKKAYELSVLCDCEIALIIFNSANRLFQYASTDMDRVLLKYTEYSEPHESRTNTDILETLKRRGVGLDGPE-    99
PonA_MEF2B   MGRKKIQISRILDQRNRQVTFTKRKFGLMKKAYELSVLCDCEIALIIFNSANRLFQYASTDMDRVLLKYTEYSEPHESRTNTDILETLKRRGIGLDGPE-    99
HomS_MEF2B   MGRKKIQISRILDQRNRQVTFTKRKFGLMKKAYELSVLCDCEIALIIFNSANRLFQYASTDMDRVLLKYTEYSEPHESRTNTDILETLKRRGIGLDGPE-    99
CalJ_MEF2B   MGRKKIQISRILDQRNRQVTFTKRKFGLMKKAYELSVLCDCEIALIIFNSANRLFQYASTDMDRVLLKYTEYSEPHESRTNTDILETLKRRGIGLDGPE-    99
AilM_MEF2B   MGRKKIQISRILDQRNRQVTFTKRKFGLMKKAYELSVLCDCEIALIIFNSANRLFQYASTDMDRVLLKYTEYSEPHESRTNTDILETLKRRGVGLDGPE-    99
MusM_MEF2B   MGRKKIQISRILDQRNRQVTFTKRKFGLMKKAYELSVLCDCDIALIIFNSAQRLFQYASSDMDRVLLKYTEYSEPHESRTNADILQTLKRRGVGLDGPE-    99
RatN_MEF2B   MGRKKIQISRILDQRNRQVTFTKRKFGLMKKAYELSVLCDCDIALIIFNSAQRLFQYASSDMDRVLLKYTEYSEPHESRTNADILQTLKRRGVGLDGPE-    99
BosT_MEF2B   MGRKKIQISRILDQRNRQVTFTKRKFGLMKKAYELSVLCDCEIALIIFNSSNRLFQYASTDMDRVLLKYTEYSEPHESRTITDILETLKRRGVGLDGPE-    99
MonD_MEF2B   MGRKKIQISRILDQRNRQVTFTKRKFGLMKKAYELSVLCDCEIALIIFNSANRLFQYASTDMDRVLLKYTEYSEPHESRTNTDILETLKRKGLGLESQE-    99
OrnA_MEF2B   MGRKKIQISRILDQRNRQVTFTKRKFGLMKKAYELSVLCDCEIALIIFNSANRLFQYASTDMDKVLLKYTEYSEPHESRTNTDILETLKRKGLGLESQE-    99
DroMel_MEF2  MGRKKIQISRITDERNRQVTFNKRKFGVMKKAYELSVLCDCEIALIIFSSSNKLYQYASTDMDRVLLKYTEYNEPHESLTNKNIIEKENKNGVMSPDSPE   100
DroSim_MEF2  MGRKKIQISRITDERNRQVTFNKRKFGVMKKAYELSVLCDCEIALIIFSSSNKLYQYASTDMDRVLLKYTEYNEPHESLTNKNIIEKENKNGVMSPDSPE   100
DroAna_MEF2  MGRKKIQISRITDERNRQVTFNKRKFGVMKKAYELSVLCDCEIALIIFSSSNKLYQYASTDMDRVLLKYTEYNEPHESLTNKNIIEKENKNGVMSPDSPE   100
DroEre_MEF2  MGRKKIQISRITDERNRQVTFNKRKFGVMKKAYELSVLCDCEIALIIFSSSNKLYQYASTDMDRVLLKYTEYNEPHESLTNKNIIEKENKNGVMSPDSPE   100
DroGri_MEF2  MGRKKIQISRITDERNRQVTFNKRKFGVMKKAYELSVLCDCEIALIIFSSSNKLYQYASTDMDRVLLKYTEYNEPHESLTNKNIIEKENKNGVMSPDSPE   100
DroMoj_MEF2  MGRKKIQISRITDERNRQVTFNKRKFGVMKKAYELSVLCDCEIALIIFSSSNKLYQYASTDMDRVLLKYTEYNEPHESLTNKNIIEKENKNGVMSPDSPE   100
DroPer_MEF2  MGRKKIQISRITDERNRQVTFNKRKFGVMKKAYELSVLCDCEIALIIFSSSNKLYQYASTDMDRVLLKYTEYNEPHESLTNKNIIEKENKNGVMSPDSPE   100
DroSec_MEF2  MGRKKIQISRITDERNRQVTFNKRKFGVMKKAYELSVLCDCEIALIIFSSSNKLYQYASTDMDRVLLKYTEYNEPHESLTNKNIIEKENKNGVMSPDSPE   100
DroVir_MEF2  MGRKKIQISRITDERNRQVTFNKRKFGVMKKAYELSVLCDCEIALIIFSSSNKLYQYASTDMDRVLLKYTEYNEPHESLTNKNIIEKENKNGVMSPDSPE   100
DroWil_MEF2  MGRKKIQISRITDERNRQVTFNKRKFGVMKKAYELSVLCDCEIALIIFSSSNKLYQYASTDMDRVLLKYTEYNEPHESLTNKNIIEKENKNGVMSPDSPE   100
DroYak_MEF2  MGRKKIQISRITDERNRQVTFNKRKFGVMKKAYELSVLCDCEIALIIFSSSNKLYQYASTDMDRVLLKYTEYNEPHESLTNKNIIEKENKNGVMSPDSPE   100
DroPse_MEF2  MGRKKIQISRITDERNRQVTFNKRKFGVMKKAYELSVLCDCEIALIIFSSSNKLYQYASTDMDRVLLKYTEYNEPHESLTNKNIIEKENKNGVMSPDSPE   100
AnoG_MEF2    MGRKKIQISRITDERNRQVTFNKRKFGVMKKAYELSVLCDCEIALIIFSSSNKLYQYASTDMDKVLLKYTEYNEPHESLTNKNIIEKENKNGTMSPDSPE   100
CulQ_MEF2d   MGRKKIQISRITDERNRQVTFNKRKFGVMKKAYELSVLCDCEIALIIFSSSNKLYQYASTDMDKVLLKYTEYNEPHESLTNKNIIEKENKNGTMSPDSPE   100
AedA_MEF2d   MGRKKIQISRITDERNRQVTFNKRKFGVMKKAYELSVLCDCEIALIIFSSTNKLYQYASTDMDKVLLKYTEYNEPHESLTNKNIIEKENKNGTMSPDSPE   100
TriC_MEF2    MGRKKIQISRITDERNRQVTFNKRKFGVMKKAYELSVLCDCEIALIIFSSSNKLYQYASTDMDKVLLKYTEYNEPHESLTNKNIIEKEHKNGVMSPDSPE   100
ApiM_MEF2    MGRKKIQISRITDERNRQVTFNKRKFGVMKKAYELSVLCDCEIALIIFSSSNKLYQYASTDMDKVLLKYTEYNEPHESLTNKNIIEKEHKGAMSPESPEP   100
BomM_MEF2    MGRKKIQISRITDERNRQVTFNKRKFGVMKKAYELSVLCDCEIALIIFSSNNKLYQYASTDMDKVLLKYTEYNEPHESLTNRNIIEALTKKEHKNGVMSP   100
CioI_MEF2    MGRKKIQISRIGDERNRQVTFTKRKFGLMKKAYELSVLCDCEIALIIFNSSNKLFQYASTDMDKVLLKYTEYNEPHESRTNADIIEMLNKKDNKNCDSPD   100
BraF_MEF2    MGRKKIQIARIDDERNRQVTFTKRKFGLMKKAYELSVLCDCEIALIIFNSANKLFQYASTDMDKVLLKYTEYNEPHESRTNSDIVEALNKKEHKGGESPD   100
CaeB_MEF2    MGRKKIQITRIQDERNRQVTFTKRKFGLMKKAYELSVLCDCEIALIVFNSTNKLFQYASTDMDKVLLKYTEYNEPHESRTNNDIMDALNRKEGNNGGGGS   100
CaeE_MEF2    MGRKKIQITRIQDERNRQVTFTKRKFGLMKKAYELSVLCDCEIALIVFNSTNKLFQYASTDMDKVLLKYTEYNEPHESRTNNDIMEALNRKEGNQGGGNS   100
CaeR_MEF2    MGRKKIQITRIQDERNRQVTFTKRKFGLMKKAYELSVLCDCEIALIVFNSTNKLFQYASTDMDKVLLKYTEYNEPHESRTNNDIMEALNRKEGNQGGGGS   100
NasV_MEF2d   MGRKKIQISRITDERNRQVTFNKRKFGVMKKAYELSVLCDCEIALIIFSSSNKLYQYASTDMDKVLLKYTEYNEPHESLTNKNIIEALNKKEHKGAMSPE   100
SacK_MEF2    MGRKKIQISRINDERNRQVTFTKRKFGLMKKAYELSVLCDCEIALIIFNSSNKLFQYASTDMDKVLLKYTEYNEPHESRTNHDIIEALNKKENKGCESPE   100
GalG_MEF2    MGRKKIQIQRITDERNRQVTFTKRKFGLMKKAYELSVLCDCEIALIIFNHSNKLFQYASTDMDKVLLKYTEYNEPHESRTNADIIETLRKKGFNGCDSPE   100
PhyI_MEF2    MGRKKIQIKRIEDDRNRQVTFAKRKNGIFKKAMELSKLCDCEIALIVFDSNEKLYQYSSTSVDQILLKYTEYGEPYETKDNNDYEIMFGE----------    90
SchM_MEF2a   MGRKKILIKKITDERNRQVTFTKRKLGLMKKAYELSILCDCEIALIVFTSSQKLFQYASSDMDKILLRYTEFNEPHESKTNRDIAELINRKEGKFSYLSD   100
             1.......10........20........30........40........50........60........70........80........90.......100
```

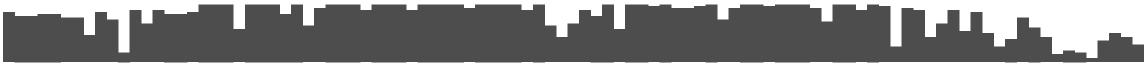

```
CanF_MEF2A   --ADDYFEHSPLSEDRFSKLNEDSDFIF---------KRGPPGLPPQNFSMSVTVPVTSPNALSYTNPGSSLVSPSLAASSTLADSSMLSPPQATLHRN-   188
MacM_MEF2A   --ADDYFEHSPLSEDRFSKLNEDSDFIF---------KRGPPGLPPQNFSMSVTVPVTSPNALSYTNPGSSLVSPSLAASSTLTDSSMLSPPQTTLHRN-   188
PanT_MEF2A   --ADDYFEHSPLSEDRFSKLNEDSDFIF---------KRGPPGLPPQNFSMSVTVPVTSPNALSYTNPGSSLVSPSLAASSTLTDSSMLSPPQTTLHRN-   188
SusS_MEF2A   P--DTYVL-TPHTEEKYKKINEEFDNMM---------RNHKPGLPPQNFSMSVTVPVTSPNALSYTNPGSSLVSPSLAASSTLADSSMLSPPQATLHRN-   187
EquC_MEF2A   P--DTYVL-TPHTEEKYKKINEEFDNMM---------RNHKPGLPPQNFSMSVTVPVTSPNALSYTNPGSSLVSPSLAASSTLADSSMLSPPQATLHRN-   187
PonA_MEF2A   --ADDYFEHSPLSEDRFSKLNEDSDFIF---------KRGPPGLPPQNFSMSVTVPVTSPNALSYTNPGSSLVSPSLAASSTLTDSSMLSPPQTTLHRN-   188
HomS_MEF2A   --ADDYFEHSPLSEDRFSKLNEDSDFIF---------KRGPPGLPPQNFSMSVTVPVTSPNALSYTNPGSSLVSPSLAASSTLTDSSMLSPPQTTLHRN-   188
CalJ_MEF2A   --ADDYFEHSPLSEDRFSKLNEDSDFIF---------KRGPPGLPPQNFSMSVTVPVTSPNALSYTNPGSSLVSPSLAASSTLTDSSMLSPPQSTLHRN-   188
AilM_MEF2A   --ADDYFEHSPLSEDRFSKLNEDSDFIF---------KRGPPGLPPQNFSMSVTVPVTSPNALSYTNPGSSLVSPSLAASSTLADSSMLSPPQATLHRN-   188
MusM_MEF2A   --ADDYFEHSPLSEDRFSKLNEDSDFIF---------KRGPPGLPPQNFSMSVTVPVTSPNALSYTNPGSSLVSPSLAASSTLADSSMLSPPPATLHRN-   188
DanR_MEF2A   P--DDCFGHSPLMDDRFGKLNEESDLMY---------KRGPTALPPQNFSMHVAVPVTNPNAMSY-NPGASLSSQSLAAAASLSDGAMLSPPQGSMHRS-   187
GalG_MEF2A   --ADDYFEHSPLSEDRFSKLNEDSDFIF---------KRGPPGLPAQNFSMSVTVPVSNPNTLTYSNPGSSLVSPSLAASSSLTSTTMLSPPQTTLHRN-   188
RatN_MEF2A   P--DTYVL-TPHTEEKYKKINEEFDNMM---------RNHKPGLPPQNFSMSVTVPVTSPNALSYTNPGSSLVSPSLAASSTLAESSMLSPPPATLHRN-   187
BosT_MEF2A   P--DTYVL-TPHTEEKYKKINEEFDNMM---------RNHKPGLPPQNFSMSVTVPVTSPSALSYTNPGSSLVSPSLAASSALADTSMLSPPQATLHRN-   187
MonD_MEF2A   --ADDYFEHSPLSEDRFSKLNEDSDFIF---------KRGPPGLPPQNFSMSVTVPVTNHNALTYTNPGSSLVSPSLAASSSLTDTSMLSPPQATMLRN-   187
XenL_MEF2A   P--DGYVL-TPHTEEKYKKINEEFDNMM---------RSHKPGLPQQTFPMSVTVPVSNPNTLPYSSPGNTMVTASLAASASLTDARMLSPPPTTLHRN-   187
OrnA_MEF2A   --ADDYFEHSPLSEDRFSKLNEDSDFIF---------KRGPPGLPPQNFSMSVTVPVTNPNALSYSNPGSSLVSPSLAASSSLADTSMLSPPQATLHRN-   188
TaeG_MEF2A   --ADDYFEHSPLSEDRFSKLNEDSDFIF---------KRGPPGLPAQNFSMSVTVPVSNPNTLTYSNPGSSLVSPSLAASSSLTDTTMLSPPQSSLHRN-   188
OryC_MEF2A   --ADDYFEHSPLSEDRFSKLNEDSDFIF---------KRGPPGLPPQNFSMSVTVPVTSPSALPYSNPGNSLVSPSLAASSTLADSSMLSPPQATLHRN-   177
CanF_MEF2C   PDADDSVGHSPESEDKYRKINEDIDLMI--------SRQRLCAVPPPNFEMPVSIPVSSHNSLVYSNPVSSLGNPNLL---PLAH--------PSLQRNS   181
MacM_MEF2C   PDADDSVGHSPESEDKYRKINEDIDLMI--------SRQRLCAVPPPNFEMPVSIPVSSHNSLVYSNPVSSLGNPNLL---PLAH--------PSLQRNS   181
PanT_MEF2C   PD-SSYAL-TPRTEEKYKKINEEFDNMI--------KSHKIPAVPPPNFEMPVSIPVSSHNSLVYSNPVSSLGNPNLL---PLAH--------PSLQRNS   179
SusS_MEF2C   PDADDSVGHSPESEDKYRKINEDLDLMI--------SRQRLCAVPPPNFEMPVSIPVSSHNSLVYSNPVSSLGNPNLL---PLAH--------PSLQRNS   181
EquC_MEF2C   PDADDSVGHSPESEDKYRKINEDIDLMI--------SRQRLCAVPPPNFEMPVSIPVSSHNSLVYSNPVSSLGNPNLL---PLAH--------PSLQRNS   181
PonA_MEF2C   PDADDSVGHSPESEDKYRKINEDIDLMI--------SRQRLCAVPPPNFEMPVSIPVSSHNSLVYSNPVSSLGNPNLL---PLAH--------PSLQRNS   181
HomS_MEF2C   PD-SSYAL-TPRTEEKYKKINEEFDNMI--------KSHKIPAVPPPNFEMPVSIPVSSHNSLVYSNPVSSLGNPNLL---PLAH--------PSLQRNS   179
CalJ_MEF2C   PDADDSVGHSPESEDKYRKINEDIDLMI--------SRQRLCAVPPPNFEMPVSIPVSSHNSLVYSNPVSSLGNPNLL---PLAH--------PSLQRNS   181
AilM_MEF2C   PDADDSVGHSPESEDKYRKINEDIDLMI--------SRQRLCAVPPPNFEMPVSIPVSSHNSLVYSNPVSSLGNPNLL---PLAH--------PSLQRNS   181
MusM_MEF2C   PDADDSVGHSPESEDKYRKINEDIDLMI--------SRQRLCAVPPPSFEMPVTIPVSSHNSLVYSNPVSTLGNPNLL---PLAH--------PSLQRNS   181
GalG_MEF2C   PDADDSVGHSPESEDKYRKINEDIDLMI--------SRQRLCAVPPPNFEMPVSIPVSNHSSLVYSNPVSSLGNPNLL---PLAH--------PSLQRNS   181
RatN_MEF2C   PDADDSVGHSPESEDKYRKINEDIDLMI--------SRQRLCAVPPPNFEMPVTIPVSSHNSLVYSNPVSSLGNPNLL---PLAH--------PSLQRNS   181
BosT_MEF2C   PDADDSVGHSPESEDKYRKINEDIDLMI--------SRQRLCAVPPPNFEMPVSIPVSSHNSLVYSNPVSSLGNPNLL---PLAH--------PSLQRNS   181
XenT_MEF2C   PDADDSVGHSPESEDKYRKINEDIDLMI--------SRQRLCAVPPPNFEMPVSIPVSNPNSLAYSNPAVSLGNHNLL---PLSH--------PSLHRNS   181
MonD_MEF2C   PDADDSVGHSPESEDKYRKINEDIDLMI--------SRQRLCAVPPPNFEMPVSIPVSNHNSLVYSNPVSSLGNPNLL---PLAH--------PSLQRNS   181
XenL_MEF2C   PDADDSVGHSPESEDKYRKINEDIDLMI--------SRQRLCAVPPPNFEMPVSIPVSNPNSLIYSNPAGSLGNHNLL---PLSH--------PSLHRNS   181
OrnA_MEF2C   PDADDSVGHSPESEDKYRKINEDIDLMI--------SRQRLCAVPPPNFEMPVSIPVSNHNSLVYSNPVSSLGNPNLL---PLAH--------PSLQRNS   181
TaeG_MEF2C   PDADDSVGHSPESEDKYRKLNEDIDLMI--------SRQRLCAVPPPNFEMPVSIPVSNHNSLVYSNPVSSLGNPNLL---PLAH--------PSLQRNS   181
OviA_MEF2C   PDADDSVGHSPESEDKYRKINEDIDLMI--------SRQRLCAVPPPNFEMPVSIPVSSHNSLVYSNPVSSLGNPNLL---PLAH--------PSLQRNS   181
OryC_MEF2C   PDADDSVGHSPESEDKYRKINEDIDLMI--------SRQRLCAVPPPNFEMPVSIPVSSHNSLVYSNPVSSLGNPNLL---PLAH--------PSLQRNS   181
DanR_MEF2Ca  IDAEDS-GHSPESEDKYRKINEDIDLMI--------SRQRLCAIPQSNYDMPVSIPVSNPNSLIYGHPGASLGNPNLL---PLTH--------PSLQRNS   180
DanR_MEF2Cb  PDADDSVGHSPESKDKYREINDDIDLMI--------SRQRICAVPSSTYDM--TIPVNNQ---IY--PG----NHNLL---PLAH--------HGLQRNS   170
CanF_MEF2D   PDGEDSLEQSPLLEDKYRRASEELDGLF---------RRYGSAVPAPNFAMPVTVPVSNQSSLQFSNPSGSLVTPSLVTSSLTDPRLLSPQQ-PALQRNS   190
MacM_MEF2D   PDGEDSLEQSPLLEDKYRRASEELDGLF---------RRYGSAVPAPNFAMPVTVPVSNQSSLQFSNPSGSLVTPSLVTSSLTDPRLLSPQQ-PALQRNS   190
PanT_MEF2D   PDGEDSLEQSPLLEDKYRRASEELDGLF---------RRYGSAVPAPNFAMPVTVPVSNQSSLQFSNPSGSLVTPSLVTSSLTDPRLLSPQQ-PALQRNS   190
SusS_MEF2D   PDGEDSLEQSPLLEDKYRRASEELDGLF---------RRYGSAVPAPNFAMPVTVPVSNQSSLQFSNPSGSLVTPSLVTSSLTDPRLLSPQQ-PALQRNS   190
EquC_MEF2D   PDGEDSLEQSPLLEDKYRRASEELDGLF---------RRYGSAVPAPNFAMPVTVPVSNQSSLQFSNPSGSLVTPSLVTSSLTDPRLLSPQQ-PALQRNS   190
PonA_MEF2D   PDGEDSLEQSPLLEDKYRRASEELDGLF---------RRYGSAVPAPNFAMPVTVPVSNQSSLQFSNPSGSLVTPSLVTSSLTDPRLLSPQQ-PALQRNS   190
HomS_MEF2D   PDGEDSLEQSPLLEDKYRRASEELDGLF---------RRYGSTVPAPNFAMPVTVPVSNQSSLQFSNPSGSLVTPSLVTSSLTDPRLLSPQQ-PALQRNS   190
CalJ_MEF2D   PDGEDSLEQSPLLEDKYRRASEELDGLF---------RRYGSAVPAPNFAMPVTVPVSNQSSLQFSNPSGSLVTPSLVTSSLTDPRLLSPQQ-PALQRNS   190
AilM_MEF2D   PDGEDSLEQSPLLEDKYRRASEELDGLF---------RRYGSAVPAPNFAMPVTVPVSSQSSLQFSNPSGSLVTPSLVTSSLTDPRLLSPQQ-PALQRNS   185
MusM_MEF2D   PDGEDSLEQSPLLEDKYRRASEELDGLF---------RRYGSSVPAPNFAMPVTVPVSNQSSMQFSNPSSSLVTPSLVTSSLTDPRLLSPQQ-PALQRNS   190
DanR_MEF2D   PEE--PFSLTPRTEEKYKKIDEEFDKMM---------QSYRSTVPAPTFSMPVTVPVSNQSSPLPFSNPS-SLVTTSFVTSTLTDPRLLSPQQ-PQLQRNT   187
GalG_MEF2D   PDGDDSIDQSPLMEDKYRKGSEDLDIILF--------KRYGSAVPTPNFAMPVTVPVTNQNTLQFSSPGSSLVTQSLVTSSLTDPRLLSPQQ-PTLQRNT   190
RatN_MEF2D   PDGEDSLEQSPLLEDKYRRASEELDGLF---------RRYGSSVPAPNFAMPVTVPVSNQSSMQFSNPSSSLVTPSLVTSSLTDPRLLSPQQ-PALQRNS   190
BosT_MEF2D   PDGEDSLEQSPLLEDKYRRASEELDGLF---------RRYGSAVPAPNFAMPVTVPVSSQSSLQFSNPSGSLATPSLVTSSLTDPRLLSPQQ-PALQRSS   190
XenT_MEF2D   PDGDDSIDQSPLMEDKYRKASEDLDIILF--------KRYGSAVPAPNFAMPVTVPVTNQNALQFSNPGGSLVTQSLMTSSLTDPRLLSPPQ-PALQRNT   190
MonD_MEF2D   PDGEDSLEQSPLLEDKYRPASEELDGLF---------RRYGSAVPAPNFAMPVTVPVSNQNSLQFSNPGGSLVTPSLVTSSLTDPRLLSPQQ-PALQRNS   190
TaeG_MEF2D   PDGDDSIDQSPLMEDKYRKGSEDLDIILF--------KRYGSTVPAPNFAMPVTVPVTNQNTLQFSNPGSSLVTQSLVTSSLTDPRLLSPQQ-PALQRNT   190
OryC_MEF2D   PDGEDSLEQSPLLEDKYRRASEELDGLF---------RRYGSAVPAPNFAMPVTVPVSNQSSLQFSNPSGSLVTPSLVTSSLTDPRLLSPQQ-PALQRNS   190
XenL_MEF2Db  PDGDDSIDQSPLMEDKYRKTSEDLDIILF--------KRYGSAVPAPNFAMPVTVPVTNQNSLHFSNPGGSLVTQSLMTSSLTDPRLLSPPQ-SALQRNT   190
XenL_MEF2Da  PDGDDSIDQSPLMEDKYRKSSEDLDIILF--------KRYGSAVPAPNFAMPVTVPVTNQNALHFSNPGGSLITQSLMTSSLTDPRLLSPPQ-PSLQRNT   190
CanF_MEF2B   ----------LEPDEGPEGPGEKLRRLA--------GDGGDPALPR--------------------------------------PRL--YPAAPTMPSPD   141
MacM_MEF2B   ----------MEPDEGPEEPGEKFRRLA--------GEGGDPALPR--------------------------------------PRL--YPAAPAMPSPD   141
PanT_MEF2B   ----------LEPDEGPEEPGEKFRRLA--------GEGGDPALPR--------------------------------------PRL--YPAAPAMPSPD   141
EquC_MEF2B   ----------LDPDEGPEGPGEKLRRLA--------GDGGDPALPR--------------------------------------PRL--YPAAPTMPSPD   141
PonA_MEF2B   ----------LEPDEGPEEPGEKFRRLA--------GEGGDPALPR--------------------------------------PRL--YPAAPAMPSPD   141
HomS_MEF2B   ----------LEPDEGPEEPGEKFRRLA--------GEGGDPALPR--------------------------------------PRL--YPAAPAMPSPD   141
CalJ_MEF2B   ----------LEPDEGPEEPGEKFRRLA--------GEGGDPALPR--------------------------------------PRL--YPAAPAMPSPD   141
AilM_MEF2B   ----------LEPDEGPEGPGEKLRRLA--------GDGGDPTLPR--------------------------------------PRL--YPAAPTMPSPD   141
MusM_MEF2B   ----------LDMEEGPEGPGEKLLRTL--------GGDRGSASPP--------------------------------------PRI--YPVAPAMSVSE   141
RatN_MEF2B   ----------LDMEEGPEGPGEKLLRTL--------GGDRGLVSPP--------------------------------------PRI--YPVAPAMSVSE   141
BosT_MEF2B   ----------LEPDEGLEGPGEKLRRLA--------GDGGDPALPR--------------------------------------PRL--YPAAPTMPSPD   141
MonD_MEF2B   ----------LELEEGLVEPGEKLRRLG--------GDGADLALVR--------------------------------------PRF--YSATPALPSPD   141
OrnA_MEF2B   ----------LELDEGPAEPAEKLRRL---------SDGVDLSLTR--------------------------------------QRL--YSAAPPLPSHN   140
DroMel_MEF2  AET--DYTLTPRTEAKYNKIDEEFQNMMQRNQMAIGGAGAPRQLPNSSYTLPVSVPVPGSYGDNLLQASPQMSHTNISPRPSSSETDSIYPSGSMLEM-S   197
DroSim_MEF2  AET--DYTLTPRTEAKYNKIDEEFQNMMQRNQMAIGGAGAPRQLPNSSYTLPVSVPVPGSYGDNLLQASPQMSHTNISPRPSSSETDSVYPSGSMLEM-S   197
DroAna_MEF2  AET--DYTLTPRTEAKYNKIDEEFQNMMQRNQMAIGGAGAPRQLPNSSYTLPVSVPVPGSYGENLLQASPQMSHTNISPRPSSSETDSVYPSGSMLEM-S   197
DroEre_MEF2  AET--DYTLTPRTEAKYNKIDEEFQNMMQRNQMAIGGAGATRQLPNSSYTLPVSVPVPGSYGDNLLQASPQMSHTNISPRPSSSETDSVYPSGSMLEM-S   197
DroGri_MEF2  AEA--DYTLTPRTEAKYNKIDEEFQNMMQRNQMAI-GA-PNRPMPNTSYTLPVSVPVPGSYGENLLQASPQMSHTNISPRPSSSETDSVYPSGTMLEM-S   195
DroMoj_MEF2  AEA--DYTLTPRTEAKYNKIDEDFQNMMQRSQMNIGGA-PNRPMPNASYTLPVSVPVPGSYGENLLQASPQMSHTNISPRPSSSETDSVYPSGTMLEM-S   196
DroPer_MEF2  AET--DYTLTPRTEAKYNKIDEEFQNMMQRNQMAIGVTGAPRQLPNSSYTLPVSVPVPGSYGENLLQASPQMSHTNISPRPSSSETDSVYPSGSMLEM-S   197
DroSec_MEF2  AET--DYTLTPRTEAKYNKIDEEFQNMMQRNQMAIGGAGAPRQLPNSSYTLPVSVPVPGSYGDNLLQASPQMSHTNISPRPSSSETDSVYPSGSMLEM-S   197
DroVir_MEF2  AEA--DYTLTPRTEAKYNKIDEEFQNMMQRNQMALSGGAPNRAMPNTSYTMPVSVPVPGSYGENLLQASPQMSHTNISPRPSSSETDSVYPSGTMLEM-S   197
DroWil_MEF2  AEA--DYTLTPRTEAKYNKIDEEFQNMMQRNQMAIGGPGAPRQLPNTSYTLPVSVPVPGSYGENMLQASPQMSHTNISPRPSSSETDSVYPSGSMLEM-S   197
DroYak_MEF2  AET--DYTLTPRTEAKYNKIDEEFQNMMQRNQMAIGGAGGPRQLPNSSYTLPVSVPVPGSYGDNLLQASPQMSHTNISPRPSSSETDSVYPSGSMLEM-S   197
DroPse_MEF2  AET--DYTLTPRTEAKYNKIDEEFQNMMQRNQMAIGVTGAPRQLPNSSYTLPVSVPVPGSYGENLLQASPQMSHTNISPRPSSSETDSVYPSGSMLEM-S   197
AnoG_MEF2    PED--SYTLTPRTEAKYNKIDEDFQIMMQRNQQLT-AGGARPNMATNSYSLPVSVPVIGTYNDSMLQSSPQMAHNSISPRPSSSETDSVYPSGGILEM-S   196
CulQ_MEF2d   PED--SYTLTPRTEAKYNKIDEDFQIMMQRNQQLS-AAGGGVGMSGNSYSLPVSVPVIGTYNDSMLQSSPQMAHNSISPRPSSSETDSVYPSGGLLEM-S   196
AedA_MEF2d   PED--SYTLTPRTEAKYNKIDEDFQIMMQRNQQLS-ASG-RVNMAGNSYSLPVSVPVIGTYNDSMMQSSPQMAHNSISPRPSSSETDSVYPSGGILEM-S   195
TriC_MEF2    PET--EYQLTPRTEAKYSKIDEEFQMMLQRNQ----INGQRVSMSSSNYTQPVSVPVNISYGDSLLQSSPQMAHTSISPRPSSSETDSVYPSGGMPEM-S   193
ApiM_MEF2    DAI--EYNLTPRTEAKYTKIDEEFQLMMQRHQ----HNGTRAMGQSNYSLPVSVPV-NSYGESLLGSSPQIAHTSISPRPSSSETDSVYPPGGMLEM-S   191
BomM_MEF2    DSPEAEYNLTPRTEAKYSKIDEEFQMMMQRNQ----LNGSRVGVGVTGSNYNLPVSVPVGNYDQSLLQASPQMHTSISPRPSSSETDSVYPSGGMLEM-S   195
CioI_MEF2    VDHQ-AQLPTPGTLQRYETINKQFDDMIN--------SMRPPP---QQSSMPVTVPVTGIHPLHGYPSPIGMHQIQHE----------QPHHPPNRRS-P   177
BraF_MEF2    DQDVEYNLLTPRTEAKYQKINEEFDRMMQ--------MNMRVTNMPPQMPVAVPVTQQQQNNILGYGAG-----------------LLQPQQIPAR-N   172
CaeB_MEF2    DDDSPGPSISPAIQIPIPAVN---------------------------------------------------------------GHNSNQNNSV-A   132
CaeE_MEF2    DDESPGPSTSPVIQITMPAV----------------------------------------------------------------VNGHSSNNSV-A   131
CaeR_MEF2    DDESPGPSTSPAIQIPIPTINGH------------------------------------------------------------TSNQSNSV-A   132
NasV_MEF2d   PEPDQYNSLTPRTEAKYAKIDEDFQMLI-------------------------------------RNQHNGSRVVYPPGGMLEM-S   148
SacK_MEF2    PGDE-PYQLTPRTEAKYSKINEEFELMMHSN-----TVMPRGSIRNYPQNMPVSIPVSQANNMTGYSSNASSSM-----------LQPQHTLGRN-S   179
GalG_MEF2    PDGDDSIDQSPLMEDKYRKGSEDLDIILF----------KRYGSAVPTFAMPVTVPVTNQNTLQFSSPGSSLVTQSLVTSSLL-----SPQQPTLQRN-T   183
PhyI_MEF2    ---------------------------KKKQLQQAAKEAAAAAAGANAEAANFNMGHGSTSNG-----------AAVQSYQTMHGGMNGASLGLTN   149
SchM_MEF2a   GDAVSNNNDPFFSNTTDQLSEADVSIRIGTKTPITSSVSMALEFPGSISDIPKHIPGITHSVTPESITDQHMNATYAPSSSMHFTTSAEREKKAENLMYH   200
             .......110.......120.......130.......140.......150.......160.......170.......180.......190.......200
```

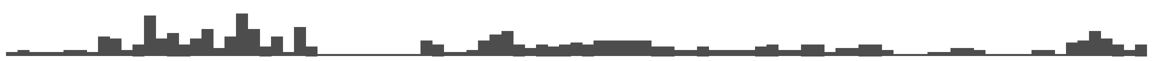

```
CanF_MEF2A   VSPGVPQRPPSTGSAGGMLSTSDLTVPNGAGSSPVGNGFVNSRASPNLIGTT--GANS--LGKVMPTKSPPPP-GGGNLGMNSRK-PDLRVVIPPTSKGM   282
MacM_MEF2A   VSPGAPQRPPSTGNAGGMLSTTDLSVPNGAGSSPVGNGFVNSRASPNLIGTT--GANS--LGKVMPTKSPPPP-GGGNLGMNSRK-PDLRVVIPPSSKGM   282
PanT_MEF2A   VSPGAPQRPPSTGNAGGMLSTTDLTVPNGAGSSPVGNGFVNSRASPNLIGAT--GANS--LGKVMPTKSPPPP-GGGNLGMNSRK-PDLRVVIPPSSKGM   282
SusS_MEF2A   VSPGAPQRPPSTGSAGGMLSTSDLTVPNGAGSSPVGNGFVNSRASPNLVGTT--GANS--LGKVMPTESPPPP-GGGNLGMNSRK-PDLRVVIPPSSKGM   281
EquC_MEF2A   VSPAAPQRPPSTGSAGGMLSTSDLTVPNGAGSSPVGNGFVNSRASPNLIGTT--GANS--LGKVMPTKSPPPP-GGGNLGMNNRK-PDLRVVIPPSSKGM   281
PonA_MEF2A   VSPGAPQRPPSTGNAGGMLSTTDLIVPNGAGSSPVGNGFVNSRASPNLIGAT--GANS--LGKVMPTKSPPPP-GGGNLGMNSRK-PDLRVVIPPSSKGM   282
HomS_MEF2A   VSPGAPQRPPSTGNAGGMLSTTDLTVPNGAGSSPVGNGFVNSRASPNLIGAT--GANS--LGKVMPTKSPPPP-GGGNLGMNSRK-PDLRVVIPPSSKGM   282
CalJ_MEF2A   VSPGAPQRPPSTGNAGGMLSTTDLTVPNGAGSSPVGNGFVNSRASPNLIGAT--GANS--LGKVMPTKSPPPP-GGGNLGMNSRK-PDLRVVIPPSSKGM   282
AilM_MEF2A   VSPGVPQRPPSTGSAGGMLSTSDLTVPNGAGSSPVGNGFVNSRASPNLIGTT--GANS--LGKVMPTKSPPPP-GGGSLGMNSRK-PDLRVVIPPSSKGM   282
MusM_MEF2A   VSPGAPQRPPSTGSASGMLSTTDLTVPNGAGNSPVGNGFVNSRASPNLIGNT--GANS--LGKVMPTKSPPPP-GGGSLGMNSRK-PDLRVVIPPSSKGM   282
DanR_MEF2A   VVP--PQRPPSTGST-------------------GNGFVNPRGSPGLLGTP--SGNG--LGKVMPAKSPPPP--GGNMGIGSRK-PDLRVVIPPSSKGM   258
GalG_MEF2A   VSPGAPQRPPSTGNAGGILGTTDLTVPNGAGTSPVGNGVWNSRASPSLLGTA--GGNG--LGKVMPTKSPPPP-GGGGLGMNNRK-PDLRVVIPPSSKGM   282
RatN_MEF2A   VSPGAPQRPPSTGSAGGMLSTTDLTVPNGAGNSPVGNGFVNSRASPNLIGNT--GANS--VGKVMPTKSPPPP-GGGSVGMNSRK-PDLRVVIPPSSKGM   281
BosT_MEF2A   VSPGAPQRPPSTGSAGGMLSTSDLTVPNGAGSSPVGNGFVNSRASPNLIGTT--GANS--LGKVMPTKSPPPP-GGGSLGMNSRK-PDLRVVIPPSSKGM   281
MonD_MEF2A   VSPGAPQRPPSTGNTGGMLSTADLTVPNGSGNSPVGNGFVNSRASPNLLVST--GGSN--LGKVMPTKSPPPP-GNGNLGMNNRK-PDLRVVIPPSSKGM   281
XenL_MEF2A   VFPGYPSEPPSTGNAGVMLCSSDLSVPNGAGTSPVGNGFVNPRASPSHLGPT--GGNV--LGKVMPTKSPPPP--GGNLVMNSRK-PDLRVVIPPSSKGM   280
OrnA_MEF2A   VSPGAPQRPPSTGNAGGMLSTADLTVPNGAGNSPVGNGFVNSRASPNLLGTS--GGNS--LGKVMPTKSPPPP-GGGNLGMNNRK-PDLRVVIPPSSKGM   282
TaeG_MEF2A   VSPGAPQRPPSAGSSGGMLGTADLSVPNGAGTSPVGNGFVNSRASPSLLGTS--GGNG--LGKVMPTKSPPPP-GGGNLGMNNRK-PDLRVVIPPSSKGM   282
OryC_MEF2A   VSPGAPQRPPSTGNAGGMLSTTDLTMPNGAGGSPVGNGFVNSRASPNLIGTT--GANS--LGKVMPTKSPPPP-GGGNLGMNSRK-PDLRVVIPPSSKGM   271
CanF_MEF2C   MSPG-VTHRPPSAGNTGGLMGGDLT--SGAGTS-AGNGYGNPRNSPGLLVSP--GN----LNKNMQAKSPPP----MNLGMNNRK-PDLRVLIPPGSKNT   266
MacM_MEF2C   MSPG-VTHRPPSAGNTGGLMGGDLT--SGAGTS-AGNGYGNPRNSPGLLVSP--GN----LNKNMQAKSPPP----MNLGMNNRK-PDLRVLIPPGSKNT   266
PanT_MEF2C   MSPG-VTHRPPSAGNTGGLMGGDLT--SGAGTS-AGNGYGNPRNSPGLLVSP--GN----LNKNMQAKSPPP----MNLGMNNRK-PDLRVLIPPGSKNT   264
SusS_MEF2C   MSPG-VTHRPPSAGNTGGLMGGDLT--SGAGTS-AGNGYGNPRNSPGLLVSP--GN----LNKNMQAKSPPP----MNLGMNNRK-PDLRVLIPPGSKNT   266
EquC_MEF2C   MSPG-VTHRPPSAGNTGGLMGGDLT--SGAGTS-AGNGYGNPRNSPGLLVSP--GN----LNKNMQAKSPPP----MNLGMNNRK-PDLRVLIPPGSKNT   266
PonA_MEF2C   MSPG-VTHRPPSAGNTGGLMGGDLT--SGAGTS-AGNGYGNPRNSPGLLVSP--GN----LNKNMQAKSPPP----MNLGMNNRK-PDLRVLIPPGSKNT   266
HomS_MEF2C   MSPG-VTHRPPSAGNTGGLMGGDLT--SGAGTS-AGNGYGNPRNSPGLLVSP--GN----LNKNMQAKSPPP----MNLGMNNRK-PDLRVLIPPGSKNT   264
CalJ_MEF2C   MSPG-VTHRPPSAGNTGGLMGGDLT--SGAGTS-AGNGYGNPRNSPGLLVSP--GN----LNKNMQAKSPPP----MNLGMNNRK-PDLRVLIPPGSKNT   266
AilM_MEF2C   MSPG-VTHRPPSAGNTGGLMGGDLT--SGAGTS-AGNGYGNPRNSPGLLVSP--GN----LNKNMQAKSPPP----MNLGMNNRK-PDLRVLIPPGSKNT   266
MusM_MEF2C   MSPG-VTHRPPSAGNTGGLMGGDLT--SGAGTS-AGNGYGNPRNSPGLLVSP--GN----LNKNIQAKSPPP----MNLGMNNRK-PDLRVLIPPGSKNT   266
GalG_MEF2C   MSPG-VTHRPPSAGNTGGLMGGDLT--TGAGTS-AGNGYGNPRNSPGLLVSP--GN----LNKNMQAKSPPP----MNLGMNNRK-PDLRVLIPPGSKNT   266
RatN_MEF2C   MSPG-VTHRPPSAGNTGGLMGGDLT--SGAGTS-AGNGYGNPRNSPGLLVSP--GN----LNKNIQAKSPPP----MNLGMNNRK-PDLRVLIPPGSKNT   266
BosT_MEF2C   MSPG-VTHRPPSAGNTGGLMGGDLT--SGAGTS-AGNGYGNPRNSPGLLVSP--GN----LNKNMQAKSPPP----MNLGMNNRK-PDLRVLIPPGSKNT   266
XenT_MEF2C   MSPG-VTHRPPSAGNTGGLMGGDLT--TGAGTS-AGNGYGNHRNSPGLLVSP--GN----LNKNMQTKSPPP----MNLGMNNRK-PDLRVLIPPGSKNT   266
MonD_MEF2C   MSPG-VTHRPPSAGNTGGLMGGDLT--TGAGTS-AGNGYGNPRNSPGLLVSP--GN----LNKNMQAKSPPP----MNLGMNNRK-PDLRVLIPPGSKNA   266
XenL_MEF2C   MSPG-VTHRPPSA---GGLMGGDLT--TGAGTS-AGNGYGNHRNSPGLLVSP--GN----LNKNMQTKSPPP----MNLGMNNRK-PDLRVLIPPGSKNT   263
OrnA_MEF2C   MSPG-VTHRPPSAGNTGGLMGGDLT--TGAGTS-AGNGYGNPRNSPGLLVSP--GN----LNKNMQAKSPPP----MNLGMNNRK-PDLRVLIPPGSKNT   266
TaeG_MEF2C   MSPG-VTHRPPSAGNTGGLMGGDLT--TGAGTS-AGNGYGNPRNSPGLLVSP--GN----LNKNMQAKSPPP----MNLGMNNRK-PDLRVLIPPGSKST   266
OviA_MEF2C   MSPG-VTHRPPSAGNTGGLMGGDLA--SGAGTS-AGNGYGNPRNSPGLLVSP--GN----LNKNMQAKSPPP----MNLGMNNRK-PDLRVLIPPGSKNT   266
OryC_MEF2C   MSPG-VTHRPPSAGNTGGLMGGDLT--SGAGTS-AGNGYGNPRNSPGLLVSP--GN----LNKNMQAKSPPP----MNLGMNNRK-PDLRVLIPPGSKSA   266
DanR_MEF2Ca  MSPG-VTHRPPSAGNTGGLMAADLG--SGAGTS-TGNGYGNHRNSPGLLVSP--GN----MSKNLQAKSPPS----MNMSL-SRK-PDLRVLIPPGAKNT   264
DanR_MEF2Cb  MSPG-VTHRPPSA---GGLMGTDLS--TGAGTS-AGNGYGNHRNSPGLLVSS--GG----MNKSMQAKSPPP----VNLGMNSRK-PDLRVLIPPGAKNN   252
CanF_MEF2D   VSPG-LPQRPASA---GAMLGGELNSANGACPSPVGNGYVSARASPGLLPVAN-GNS---LNKGIPAKSPPPPTHSAQLGAPSRK-PDLRVITSQGGKGL   281
MacM_MEF2D   VSPG-LPQRPASA---GAMLGGDLNSANGACPSPVGNGYVSARASPGLLPGAN-GNS---LNKSFPAKSPPPPTHGTQLGAPSRK-PDLRVITSQAGKGL   281
PanT_MEF2D   VSPG-LPQRPASA---GAMLGGDLNSANGACPSPVGNGYVSARASPGLLPVAN-GNS---LNKVIPAKSPPPPTHSTQLGAPSRK-PDLRVITSQAGKGL   281
SusS_MEF2D   VSPG-LPQRPASA---GAMLGGDLNSANGACPSPVGNGYVSARASPGLLPVAN-GNS---LNKVIPAKSPPPPTHSAQLGAPSRK-PDLRVITSQGGKGL   281
EquC_MEF2D   VSPG-LPQRPASA---GAMLGGDLTSANGACPSPVGNGYVSARASPGLLPVAN-GNS---LNKVIPAKSPPPPTHSAQLGAPSRK-PDLRVITSQGGKGL   281
PonA_MEF2D   VSPG-LPQRPASA---GAMLGGDLNSANGACPSPVGNGYVSARASPGLLPVAN-GNS---LNKVIPAKSPPPPTHSTQLGAPSRK-PDLRVITSQAGKGL   281
HomS_MEF2D   VSPG-LPQRPASA---GAMLGGDLNSANGACPSPVGNGYVSARASPGLLPVAN-GNS---LNKVIPAKSPPPPTHSTQLGAPSRK-PDLRVITSQAGKGL   281
CalJ_MEF2D   VSPG-LPQRPASA---GAMLGGDLNSANGACPSPVGNGYVSARASPGLLPVAN-GNS---LNKVIPAKSPPPPTHSTQLGAPSRK-PDLRVITSQAGKGL   281
AilM_MEF2D   VSPG-LPQRPASA---GAMLGGDLNSANGACPSPVGNGYGSARASPGLLPVAN-GNS---LNKGIPAKSPPPPTHSAQLGAPSRK-PDLRVITSQGGKGL   276
MusM_MEF2D   VSPG-LPQRPASA---GAMLGGDLNSANGACPSPVGNGYVSARASPGLLPVAN-GNS---LNKVIPAKSPPPPTHNTQLGAPSRK-PDLRVITSQGGKGL   281
DanR_MEF2D   VSPG-LPQRPASA---GALLGGDLNNSNGACPSPVANGYVSARASPGLLSVSN-GNS---MGKVVQAKSPPPP--STQMA-NSRK-PDLRVITSQSSKGL   275
GalG_MEF2D   VSPG-LPQRPASA---GAMLGGDLNNTNGACPSPVGNGYVSARASPGLLPVSN-GST---LGKIIPAKSPPPPPHSTQLASNSRK-PDLRVITSQSGKGL   281
RatN_MEF2D   VSPG-LPQRPASA---GAMLGGDLNSANGACPSPVGNGYVSARASPGLLPVAN-GNG---LNKVIPAKSPPPPTHNTQLGAPSRK-PDLRVITSQGGKGL   281
BosT_MEF2D   VSPG-LPQRPASA---GAMLGGDLSSANGACPSPVGNGYVSARASPGLLPVAN-GNS---LNKVIPAKSPPPPAHSAQLGAPSRK-PDLRVITSQGGKGL   281
XenT_MEF2D   VSPG-LPQRPASA---GAMLGGELNNSNGTCPSPVGNGYISARASPGLIPVSN-GNS---LGKVIPAKSPPPPNQNSQLGANSRK-PDLRVITSQGGKGL   281
MonD_MEF2D   VSPG-LPQRPASA---GAMLGGDLNSANGACPSPVGNGYVSARASPGLLPVAN-GNS---LSKVIPAKSPPPPTHSAQLGAPSRK-PDLRVITSQGGKGL   281
TaeG_MEF2D   VSPG-LPQRPASA---GAMLGGDLNNTNGACPSPVGNGYVSARASPGLLPVSN-GSS---LGKIIPAKSPPPPAHGAQLAANSRK-PDLRVITSQSGKGL   281
OryC_MEF2D   VSPG-LPQRPASA---GAMLGGDLTSANGACPSPVGNGYVSARASPGLLPVAN-GNS---LNKVIPAKSPPPPPHGAQLGAPSRK-PDLRVITSQGGKGL   281
XenL_MEF2Db  VSPG-LPQRPASA---GAMLGGELN-SNGTCPSPVGNGYISARASPGLLPVSN-GNS---MGKVIPAKSPTPPNQNNQLGVNSRK-PDLRVITSQGGKGL   280
XenL_MEF2Da  VSPG-LPQRPASA---GAMLGGELNNSNGTCPSPVGNGYISARASPGLLPVSN-GNS---LGKVIQAKSPPSPNQNSQLGANSRK-PDLRVITSQGGKGL   281
CanF_MEF2B   MVYG-ALPPPGCD-----------PSGLGEALPAQSRPSPFRPAAPKAGPP-GLA---HPLFSPSHLASKTPPPLYLAADGRR-PDLPGSLAGARGGLS   223
MacM_MEF2B   MVYG-ALPPPGCD-----------PSGLGEALPAQSRPSPFRPAAPKAGPP-GLV---HPLFSPSHLTSKTPPPLYLPTEGRR-PDLPGGLAGPRGALN   223
PanT_MEF2B   VVYG-ALPPPGCD-----------PSGLGEALPAQSRPSPFRPAAPKAGPP-GLV---HPLFSPSHLTSKTPPPLYLPTEGRR-SDLPGGLAGPRGGLS   223
EquC_MEF2B   MVYG-ALPPPGCE-----------PSGLGEALSAQSRPSPFRPAAPKAGPP-GLA---HPLFSPSHLASKTPPPLYLAADGRR-PDLPGGLAGARGGLS   223
PonA_MEF2B   VVYG-ALPPPGCD-----------PSGLGEALPAQSRPSPFRPAAPKAGPP-GLV---HPLFSPSHLTSKTPPPLYLPTEGRR-SDLPGGLAGPRGGLN   223
HomS_MEF2B   VVYG-ALPPPGCD-----------PSGLGEALPAQSRPSPFRPAAPKAGPP-GLV---HPLFSPSHLTSKTPPPLYLPTEGRR-SDLPGGLAGPRGGLN   223
CalJ_MEF2B   MVYG-ALPPPGCD-----------PSGLGEALPAQSRPSPFRPAAPKAGPP-GLA---HPLFSPSHLTSKTPPPLYLPADGRR-ADLPGGLAGPRGGLN   223
AilM_MEF2B   MVYG-ALPPPGCD-----------PSGLGEALPAQSRPSPFRPAAPKAGPP-GLA---HPLFSPSHLASKTPPPLYLAADGRR-PDLPGSLAGARGGLS   223
MusM_MEF2B   LSYR-VPPAPGCD-----------PGGLGEAPSVHSRPPHFRP-------P-GLG---HPIFSPSHLASKTPPPLYLATDGRR-PDLPPGLVGARGGLG   216
RatN_MEF2B   LSYR-VPPASGCD-----------PGGLGEAPSVHSRSPHFRP-------P-GLG---HPIFSPSHLAGKTPPPLYLATDGRR-PDLPPGLVGARGGLG   216
BosT_MEF2B   MVYG-ALPPPACE-----------PTGLGEALPAQSRPSPFRPAAPKAGPP-GLA---HPLFSPSHLASKTPPPLYLAADGRR-PDLPGGLAGTRGGLS   223
MonD_MEF2B   LAYG-AVPPPGSG-----------PEPCGEATPGQGRPSPFRPAASKAGPP-GLG---HPLFSPRALANKTPPPLYLTADGRR-SDLPGNLAAPRAGLG   223
OrnA_MEF2B   VAYG-AVPPPRTD-----------PAGLGEPSPSQSHPPPFKPAGPKPGPP-GLS---YPLFSTRALASKTPPPLYLGADARR-PDLTSSLGGARGTLS   222
DroMel_MEF2  NGYP-HSHSPLVGSPSPGPSPGI----AHHLSIKQQSPGSQNGRASNLRVVIPPTIA---PIPPNMSAPDDVGYADQRQSQTSLNT-PVVTLQTPIPALT   288
DroSim_MEF2  NGYP-HSHSPLVGSPSPGPSPGI----AHHLSIKQQSPGSQNGRASNLRVVIPPTIA---PIPPNMSAPDDGGYADQRQSQTSLNT-PVVTLQTPIPALT   288
DroAna_MEF2  NGYP-HSHSPLVGSPSPGPSPGI----AHHLSIKQQSPGSQNGRASNLRVVIPPTIA---PLPPNMSAPDDVGYGDQRQSQTSLNT-PVVTLQTPIPALT   288
DroEre_MEF2  NGYP-HSHSPLVGSPSPGPSPGI----AHHLSIKQQSPGSQNGRASNLRVVIPPTIA---PIPPNMSAPDDGGYADQRQSQTSLNT-PVVTLQTPIPALT   288
DroGri_MEF2  NGYP-HSHSPLVGSPSPGPSPGI----AHHLPIKQQSPGSQNGRASNLRVVIPPTIA---P-NMSATA-DDVAYADPRQSQTSLNT-PVVTLQTPMPPLT   284
DroMoj_MEF2  NGYP-HSHSPLVGSPSPGPSPGI----AHHLPIKQQSPGSQNGRASNLRVVIPPTIA---P-NMSATA-DDVAYADQRQSHTSLNT-PVVTLQTPMPPLA   285
DroPer_MEF2  NGYP-HSHSPLVGSPSPGPSPGI----AHHLPIKQQSPGSQNGRAANLRVVIPPTIA---TIPPNMSAPEDVAYTDQRQSQTSLNT-PVVTLQTPIPALT   288
DroSec_MEF2  NGYP-HSHSPLVGSPSPGPSPGI----AHHLSIKQQSPGSQNGRASNLRVVIPPTIA---PIPPNMSAPDDGGYADQRQSQTSLNT-PVVTLQTPIPALT   288
DroVir_MEF2  NGYP-HSHSPLVGSPSPGPSPGI----AHHLPIKQQSPGSQNGRASNLRVVIPSTIA---P-NMSATA-DDVAYADQRQSQTSLNT-PVVTLQTPMPPLA   286
DroWil_MEF2  NGYP-HSHSPLVGSPSPGPSPGI----AHHMPIKQQSPGSQNGRASNLRVVIPPSIG---P-NMS-TA-DDVAYGDHRQSQTSLNT-PVVTLQTPIPALT   285
DroYak_MEF2  NGYP-HSHSPLVGSPSPGPSPGI----AHHLSIKQQSPGSQNGRASNLRVVIPPTIA---PIPPNMSAPDDGGYADQRQSQTSLNT-PVVTLQTPIPALT   288
DroPse_MEF2  NGYP-HSHSPLVGSPSPGPSPGI----AHHLPIKQQSPGSQNGRAANLRVVIPPTIA---TIPPNMSAPEDVAYTDQRQSQTSLNT-PVVTLQTPIPALT   288
AnoG_MEF2    NGYP-HSASPLGGSPSPGPSPGIGSHQAHTIKQSPSGGSGSSGSRS-LRVVIPAPMN---P---TITA-DDISYVEHTRQQTNLNT-PVVALQTPIPGLT   286
CulQ_MEF2d   NGYP-HSASPLGGSPSPGPSPGI----VQHQSLKQNSPGSSSTRSSNLRVVIPTPMN---P---SMG-NDDMSYVEHSRQQTNLNT-PVVALQTPIPGLS   283
AedA_MEF2d   NGYP-HSASPLGGSPSPGPSP------VQHQTIKQGSPGSSNGRSSNLRVVIPTPMN---P---TIN-TDDLSYVEHSRQQSSNLNTPVVALQTPIPGLS   281
TriC_MEF2    NGYP-NSASPMDGSPSPGPSPG-----PGLSSSKQHPKQHSPGPRSNLRVVIPTALG---P---SIT-PEEVSYGEHNRTQSTLNT-PVVALQTP--GLS   277
ApiM_MEF2    NGYP-PSASPLGGSPSPGPSPALGVG-SRHSPQPPPPPPPPHPHRTNLRVVIPTPLTQ--P-----L-SEDTSY-DNGHTQSALNT-PVVALQTPSVPAG   279
BomM_MEF2    NGYP-GSASPLG-APSPSPGPAPSPHRHPHKSHAHAPPPHHSPRHNNLRVVIPSSMP---P--------------------PQDD--ISYAETPLSYS   266
CioI_MEF2    LPKS-PLARGHSPSRSPSGQMYSPEVNMISDTPSPGGNGYSGGNQPPPPQQRGPSPH----MDRTSPMPHKPMQQSPT-SPARGHR-PGLHVVIPPS---   267
BraF_MEF2    VSPG-VQPQRPPSNGVPGAMLGADLTGTSAGAGTAGNGYVNQNRASPGVMVSSAAQS-----------------------KQPS-PSLPRSGGHPTLR   245
CaeB_MEF2    SASS-ASAAAVAAAAAAVAAVEGTSSGAAAAAAALQASNAQRHHNLNLYQNLIFNPN----YTRHLPQRNDPLTSSSSAAPSSSSS-SSKHLDFPPSTSF   226
CaeE_MEF2    SAAS-ASAAAVAAAAAAVAAVEGTSSGAAAAAAALQASNAQRHHNLNLYQNLIFNPN----YTRHLNQRNDPLSSTS-VAPSSSSS---KHLDFPPSTSF   222
CaeR_MEF2    SAAS-ASAAAVAAAAAAVAAVEGTSSGAAAAAAALQASNAQRHHNLNLYQNLIFNPN----YARHLPPRNEPLPSSS-AAPSSSSS-SSKHLDFPPSTSF   225
NasV_MEF2d   NGYP-PSASPLGGSPSPGPSPSLGVSKGNPSRHSPQPPPPPHPHRGALRVVIPTPLGQ--SLAEEPSYDSGHAQSSLNTPVVALQT-PSVPPSYTSFGPT   244
SacK_MEF2    VSPV-SNMTQPQRPSSSGPPSTVLSNDSSSATSTSTGTGTTDHHLNYRKIGYSSNQE---------------YISSSVGSNYGNP-SRTSPGGTSPGGL   261
GalG_MEF2    VSPG-LPQRPASAGAMLGGDLNN----TNGACPSPVGTGYVSARASAGLLPVSNGST----LKIILAKSLPRSPHSTQLASNSRKQ-NMRVITSQSGKGL   273
PhyI_MEF2    DPDQ----------------YMLNSRSRPPTQKKRVHRGFQQLLKKEHGG-------------------------------------------------   183
SchM_MEF2a   EQDTTSGVVIGESRVNELCREIILDDETNQPFNSDLRSSSRNHIPQIHFGSQEFSTHNRGSSLIDPNMKSIVSSDHLSSFRPENIQNFRSEVQDCFNNNL   300
             .......210.......220.......230.......240.......250.......260.......270.......280.......290.......300
```

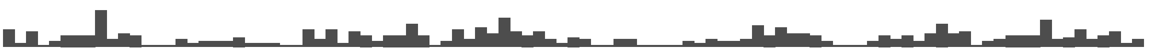

```
CanF_MEF2A    MPPLSEEEELELNTQRISSSQATQPLATPVVSVTTPSLPPQGL-VY-SAMPTAYNTDYSLTSADLSALQGFNSPGMLSLGQVSAWQQH------------   368
MacM_MEF2A    MPPL--------NTQRISSSQATQPLATPVVSVTTPSLPPQGL-VY-SAMPTAYNTDYSLTSADLSALQGFNSPGMLSLGQVSAWQQH------------   360
PanT_MEF2A    MPPLSEEEELELNTQRISSSQATQPLATPVVSVTTPSLPPQGL-VY-SAMPTAYNTDYSLTSADLSALQGFNSPGMLSLGQVSAWQQH------------   368
SusS_MEF2A    MPPLSEEEELELNTQRISSSQAPQPLATPVVSVTTPSLPQQGL-VY-SAMPTAYNTDYSLTSADLSALQGFNSPGMLSLGQVSAWQQH------------   367
EquC_MEF2A    MPPLSEEEELELNTQRISSSQATQPLATPVVSVTTPSLPPQGL-VY-SAMPTAYNTDYSLTSADLSALQGFNSPGMLSLGQVSAWQQH------------   367
PonA_MEF2A    MPPL--------NTQRISSSQATQPLATPVVSVTTPSLPPQGL-VY-SAMPTAYNTDYSLTSADLSALQGFNSPGMLSLGQVSAWQQH------------   360
HomS_MEF2A    MPPL--------NTQRISSSQATQPLATPVVSVTTPSLPPQGL-VY-SAMPTAYNTDYSLTSADLSALQGFNSPGMLSLGQVSAWQQH------------   360
CalJ_MEF2A    MPPL--------NTQRISSSQATQPLATPVVSVTTPSLPPQGL-VY-SAMPTAYNTDYSLTSADLSALQGFNSPGMLSLGQVSAWQQH------------   360
AilM_MEF2A    MPPL--------NTQRISSSQATQPLATPVVSVTTPSLPPQGL-VY-SAMPTAYNTDYSLTSADLSALQGFNSPGMLSLGQVSAWQQH------------   360
MusM_MEF2A    MPPLSEEEELELNAQRISSSQATQPLATPVVSVTTPSLPPQGL-VY-SAMPTAYNTDYSLTSADLSALQGFTSPGMLSLGQASAWQQH------------   368
DanR_MEF2A    MPPL--------NTQRISSSQSTQPLATPVVSVTTPSLAPQGL-VY-SGMPTAYNSEYSLSSAELSSLQGFGSPG-LSIGSVSAWQQH------------   335
GalG_MEF2A    MPPLTEEDELELNTQRISSSQSTQPLATPVVSVTTPSFPPAGL-VY-SAMPTAYNTDYSLTSADLSAFEGFNSPGMLSLGQVSPWQQH------------   368
RatN_MEF2A    MPPL--------NAQRISSSQATQPLATPVVSVTTPSLPPQGL-VY-SAMPTAYNTDYSLTSADLSALQGFTSPGMLSLGQASAWQQH------------   359
BosT_MEF2A    MPPL--------NTQRISSSQATQPLATPVVSVTTPSLPPQGL-VY-SAMPTAYNTDYSLTSADLSALQGFNSPGMLSLGQVSAWQQH------------   359
MonD_MEF2A    MPPLSEEDELELNSQRISSSQSTQPLATPVVSVTTPSLPPQGL-VY-SAMPTAYNTDYSLTSADLSALQGFNSPGMLSLGQVSAWQQH------------   367
XenL_MEF2A    MPPL--------NTQRVTSSQGTQPLATPIVSVATPSLAPQGL-IY-SAMPTAYNTDYPLTSADLSMLQGFNSPGILPLGQVSAWQQH------------   358
OrnA_MEF2A    MPPLSEEDELELNTQRISSSQSTQPLATPVVSVTTPSLPPQGL-VY-SAMPTAYNTDYSLTSADLSALQGFNSPGMLSLGQVSAWQQH------------   368
TaeG_MEF2A    MPPLSEEDELELNPQRISSSQSTQPLATPVVSVTTPSLPPQGL-VY-SAMPTAYNTDYSLTSADLSALQGFNSPGMLSLGQVSAWQQH------------   368
OryC_MEF2A    MPPLSEEEELELNPQRISSSQATQPLATPVVSVTTPSLPPQGL-VY-SAMPTAYNTDYSLTSADLSALQGFNSPGMLSLGQVSAWQQH------------   357
CanF_MEF2C    MPSVSEDVDLLLN-QRINNSQSAQSLATPVVSVATPTLPGQGMGGYPSAISTTYGTEYSLSSADLSSLSGFNTASALHLGSVTGWQQQ------------   353
MacM_MEF2C    MPSVSEDVDLLLN-QRINNSQSAQSLATPVVSVATPTLPGQGMGGYPSAISTTYGTEYSLSSADLSSLSGFNTASALHLGSVTGWQQQ------------   353
PanT_MEF2C    MPSVSEDVDLLLN-QRINNSQSAQSLATPVVSVATPTLPGQGMGGYPSAISTTYGTEYSLSSADLSSLSGFNTASALHLGSVTGWQQQ------------   351
SusS_MEF2C    MPSV--------N-QRINNSQSAQSLATPVVSVATPTLPGQGMGGYPSAISTTYGTEYSLSSADLSSLSGFNTASALHLGSVTGWQQQ------------   345
EquC_MEF2C    MPSVSEDVDLLLN-QRINNSQSAQSLATPVVSVATPTLPGQGMGGYPSAISTTYGTEYSLSSADLSSLSGFNTASALHLGSVTGWQQQ------------   353
PonA_MEF2C    MPSVSEDVDLLLN-QRINNSQSAQSLATPVVSVATPTLPGQGMGGYPSAISTTYGTEYSLSSADLSSLSGFNTASALHLGSVTGWQQQ------------   353
HomS_MEF2C    MPSVSEDVDLLLN-QRINNSQSAQSLATPVVSVATPTLPGQGMGGYPSAISTTYGTEYSLSSADLSSLSGFNTASALHLGSVTGWQQQ------------   351
CalJ_MEF2C    MPSVSEDVDLLLN-QRINNSQSAQSLATPVVSVATPTLPGQGMGGYPSAISTTYGTEYSLSSADLSSLSGFNTASALHLGSVTGWQQQ------------   353
AilM_MEF2C    MPSVSEDVDLLLN-QRINNSQSAQSLATPVVSVATPTLPGQGMGGYPSAISTTYGTEYSLSSADLSSLSGFNTASALHLGSVTGWQQQ------------   353
MusM_MEF2C    MPSV--------N-QRINNSQSAQSLATPVVSVATPTLPGQGMGGYPSAISTTYGTEYSLSSADLSSLSGFNTASALHLGSVTGWQQQ------------   345
GalG_MEF2C    MPSVSEDVDLLLN-QRINNSQSAQSLATPVVSVATPTLPGQGMGGYPSAISTTYGTEYSLSSADISSLSGFNTASALHLGSVTGWQQQ------------   353
RatN_MEF2C    MPSV--------N-QRINNSQSAQSLATPVVSVATPTLPGQGMGGYPSAISTTYGTEYSLSSADLSSLSGFNTASALHLGSVTGWQQQ------------   345
BosT_MEF2C    MPSVSEDVDLLLN-QRINNSQSAQSLATPVVSVATPTLPGQGMGGYPSAISTTYGTEYSLSSADLSSLSGFNTASALHLGSVTGWQQQ------------   353
XenT_MEF2C    MPSV--------N-QRINNSQSAQSLATPVVPVATPTLPGQGMGGYPTAISTTYGTEYSLSSADLSSLSGFNT-SALHLGSVTGWQQH------------   344
MonD_MEF2C    MPSVSEDVDLLLN-QRINNSQSAQSLATPVVSVATPTLPGQGMGGYPSAISTTYGTEYSLSSADLSSLSGFNTASALHLGSVTGWQQQ------------   353
XenL_MEF2C    MPSV--------N-QRINNSQSAQSLATPVVPVATPTLPGQGMGGYPTAISTTYGTEYALSSADLSSLSGFNT-SALHLGSVTGWQQH------------   341
OrnA_MEF2C    MPSVSEDVDLLLN-QRINNSQSAQSLATPVVSVATPTLPGQGMGGYPSAISTTYGTEYSLSSADLSSLSGFNTASALHLGSVTGWQQQ------------   353
TaeG_MEF2C    MPSVSEDVDLLLN-QRINNSQSAQSLATPVVSVATPTLPGQGMGGYPSAISTTYGTEYSLSSADISSLSGFNTASALHLGSVTGWQQQ------------   353
OviA_MEF2C    MPSV--------N-QRINNSQSAQSLATPVVSVATPTLPGQGMGGYPSAISTTYGTEYSLSSADLSSLSGFNTASALHLGSVTGWQQQ------------   345
OryC_MEF2C    MPSV--------N-QRINNSQSAQSLATPVVSVATPTLPGQGMGGYPSAISTTYGTEYSLSSADLSSLSGFNAASALHLGSVTGWQQQ------------   345
DanR_MEF2Ca   MPSI--------N-QRISSSQSAQSLSTPVVSVATPTLPGEVMGGYPSAINTSYGTEYSLSSGDLSSLSAFNSSASLHLGSMTGWPQN------------   343
DanR_MEF2Cb   MPSI--------N-QRINNSQSAQSLATPVVSVATPTLPGQGMGGYPSAISTSYGTEYSLSSADLSSISGFNSANTLHLGSMSGWQQH------------   331
CanF_MEF2D    MHHL-------NNAQRLGASQSTHSLTTPVVSVATPSLLSQGL--PFSSMPTAYNTDYQLSSAELSSLPTFSSPGGLSLGGVT-----------------   355
MacM_MEF2D    MHHLTEDHLDLNNAQRLGVSQSTHSLTTPVVSVATPSLLSQGL--PFSSMPTAYNTDYQLTSAELSSLPAFSSPGGLSLGNVTAWQQPQQPQQPQQPQPP   379
PanT_MEF2D    MHHLTEDHLDLNNAQRLGVSQSTHSLTTPVVSVATPSLLSQGL--PFSSMPTAYNTDYQLTSAELSSLPAFSSPGGLSLGNVTAWQQPQQPQQPQQPQPP   379
SusS_MEF2D    MHHLTEDHLDLNNAQRLGVSQSTHSLTTPVVSVATPSLLSQGL--PFSSMPTAYNTDYQLTSAELSSLPTFSSPGGLSLGNVTAWQQPQQPQQPPQPQPP   379
EquC_MEF2D    MHHLTEDHLDLNNAQRLGVSQSTHSLTTPVVSVATPSLLSQGL--PFSSMPTAYNTDYQLTSAELSSLPSFSSPGGL-----------------------   356
PonA_MEF2D    MHHLTEDHLDLNNVQRLGVSQSTHSLTTPVVSVATPSLLSQGL--PFSSMPTAYNTDYQLTSAELSSLPAFSSPGGLSLGNVTAWQQPQQPQQPQQPQPP   379
HomS_MEF2D    MHHLTEDHLDLNNAQRLGVSQSTHSLTTPVVSVATPSLLSQGL--PFSSMPTAYNTDYQLTSAELSSLPAFSSPGGLSLGNVTAWQQPQQPQQPQQPQPP   379
CalJ_MEF2D    MHHLTEDHLDLNNAQRLGVSQSTHSLTTPVVSVATPSLLSQGL--PFSSMPTAYNTDYQLTSAELSSLPAFSSPGGLSLGNVTAWQQPQQPQ--------   371
AilM_MEF2D    MHHLTEDHLDLNNAQRLGASQSTHSLTTPVVSVATPSLLSQGL--PFSSMPTAYNTDYQLSSAELSSLPTFSSPGGLQPQPQ------------------   356
MusM_MEF2D    MHHL-------NNAQRLGVSQSTHSLTTPVVSVATPSLLSQGL--PFSSMPTAYNTDYQLPSAELSSLPAFSSPAGLALGNVTAWQQPQPPQQPQPPQPP   372
DanR_MEF2D    MQLTDEEELVSENAQRLGVSQSVTQPLTTPVVSVATPSLLT-----PF-SMPTAYNTEYTLTSADLTALQAFTPSGALLPGNMTTWQQQQQPSVSQQPQQQ   369
GalG_MEF2D    MHHLTEDHLALQNTQRLGVSQATHSLTTPVVSVATPSLLTQGL--PFSAMPTAYNTARSLGGIRTHSEAERQR---------------------------   352
RatN_MEF2D    MHHL-------NNAQRLGVSQSTHSLTTPVVSVATPSLLSQGL--PFSSMPTAYNTDYQLPSAELSSLPAFSSPAGLALGNVTAWQQPQQPQQPQPPQPP   372
BosT_MEF2D    MHHLTEDHLDLNNAQRLGVSQSTHSLTTPVVSVATPSLLSQGL--PFSSMPTAYNTDYQLTSAELSSLPAFSSPGGLSLSNVTAWQQPQQPPQPQPPQQP   379
XenT_MEF2D    MHHLTEEQLEMSAQRLGGVSQAAHSLTTPVVSVATPSLLSHGL--PFSAMPTAYNTDYQLTSADLSSLSTFSSPSSLSLGNVTAWQQQQQQH-------   372
MonD_MEF2D    MHHLTEDHLDLNNAQRLGVSQSTHSLTTPVVSVATPSLLSQGL--PFSSMPTAYNTDYQLTSAELSSLPAFSSTGGLSLGNVTAWQQQQPQPPQQQPQP   379
TaeG_MEF2D    MHHLVSAHSSQN-TQRLGVSQATHSLTTPVVSVATPSLLTQGL--PFSAMPTAYNT--VHTSA-GSHLSHTTTLTVNTNPN-------------------   356
OryC_MEF2D    MHHL-------NNAQRLGVSQSTHSLTTPVVSVATPSLLSQGL--PFSSMPTAYNTDYQLTSAELSSLPAFSSPGGLSLGNVTAWQQPQQPQQPQPPQPQ   372
XenL_MEF2Db   MHHLTEEQLEMSAQRLGGVSQAAHSLTTPVVSVATPSLLSHGL--PFSAMTTAYNTDYQLTSADLSSLSSFSSPGSLSLGNVTAWQQHHQQQQH------   372
XenL_MEF2Da   MHHLTEEQLEMSVQRLGGVSQATHSLTTPVVSVATPSLLSHGL--PFSAMSTAYNTDYQLTSADLASLSTFSSPGSLSLGNVTAWQHQQQQQQQ------   373
CanF_MEF2B    TS----------RGLYGSLQSPCSATAPGPPLGSFPFLPAGPPAA------------------------------------------------------   258
MacM_MEF2B    TS----------RSLYSGLQSPCSTATPGPPLGSFPFLPAGPAEYGLGDPPPPPGLLQPPTLAPWQPSRGDGPPAV------------------------   289
PanT_MEF2B    TS----------RSLYSGLQNPCSTATPGPPLGSFPFLPGGPPEYGLGDPPPPPGLLQPPTLAPWQPSRGDGPPAV------------------------   289
EquC_MEF2B    TS----------RGLYGSLQSPGSAAAPGPPLGSFPFLPAGPPEYGLGDPPPPPGLLQPPTLAPWQPSRGDGPPAG------------------------   289
PonA_MEF2B    TS----------RSLYSGLQNPCSTATPGPPLGSFPFLPGGPPEYGLGDPPPPPGLLQPPTLAPWQPSRGDGPPAV------------------------   289
HomS_MEF2B    TS----------RSLYSGLQNPCSTATPGPPLGSFPFLPGGPPEYGLGDPPPPPGLLQPPTLAPWQPSRGDGPPAV------------------------   289
CalJ_MEF2B    TS----------RSLYSGLQSPCSSAAPGPPLGSFPFLPAGPPEYGLGDPPPPPGLLQPPTLASWQPSRGEGPPAV------------------------   289
AilM_MEF2B    TS----------RGLYGSLQSPCSATAPGPPLGSFPFLPAGPP--GM-HHPHVSFCGRHPYTCLLGHSRGDGPPAA------------------------   286
MusM_MEF2B    TS----------RSLYSGLQSP---GAPGPALGSFAFLPSGSTDCSPGDAAQGP--LQPSPWPPTRDAV-------------------------------   270
RatN_MEF2B    TS----------RSLYSGLQSP---GAPGPALGSFAFLPPGSADCSPGDAAQVP--LQPSPWPPTRDAV-------------------------------   270
BosT_MEF2B    SS----------RGLYGGLQSPCSTAAPGPPLGSFPFLPAGPPEYGLGDPPPPPGLLQPPTLAPWQPSRADGPPAT------------------------   289
MonD_MEF2B    AT----------RTLYGSLQSPSPISAPGNGLGAFPFISSSQAEYGHGDPSPHPGLLQPTALVPWQPPRGDRSPSS------------------------   289
OrnA_MEF2B    ST----------RTLYSSLQNSSPMVATGNGLGAFPFLPSGPTEYGPGEASPHPGFLQPNAMASWQPPRDNPTSL-------------------------   287
DroMel_MEF2   SYSFGAQDFSSSGVMNSADIMSLNTWHQGLVPHSSLSHLAVSNSTPPPATSPVSIKVKAEPQSPPRDLSASGHQQNSNGSTGSGGSSSSTSSNASGGAGG   388
DroSim_MEF2   SYSFGAQDFSSSGVMNSADIMSLNTWHQGLVPHSSLSHLAVSNSTPPPATSPVSIKVKAEPQSPPRDLSASGHQQNSNGSTGSGGSSSSTSSNASGGAGG   388
DroAna_MEF2   SYSFGAQDFSSSGVMGSADIMSLNTWHQSLVPHSSLSHLAVSNSTPPPATSPVSIKVKAEPQSPPRDLSASGHQQNSNGSTGSGGSSSTTSSNTSGNGGG   388
DroEre_MEF2   SYSFGAQDFSSSGVMNSADIMSLNTWHQGLVPHSSLSHLAVSNSTPPPATSPVSIKVKAEPQSPPRDLSASGHQQNSNGSTGSGGSSSSTSSNASGGGGG   388
DroGri_MEF2   SYSFGAQDFSQSGAM-SADIMSLPQWHQGLVQHTSLSHLAVSNSTPPPASSPVSIKVKAEPQSPPRDLSASGHQHNSNGSTGSGSSSSSTSSNVSTAGGV   383
DroMoj_MEF2   SYTFGAQDFSQSGAM-SADIMSLPQWHQGLVQHTSLSHLAVSNSTPPPASSPVSIKVKAEPQSPPRDLSASGHQHNSNGSTGSGSSSSSTSSNVSTAGVL   384
DroPer_MEF2   SYSFGAQDFSSSGVIGSADIMSLPTWHQGLVQHSSLSHLAVSNSTPPPATSPVSIKVKAEPQSPPRDLSASGHQQNSNGSTGSGGSSSSTSSNASAGGGG   388
DroSec_MEF2   SYSFGAQDFSSSGVMNSADIMSLNTWHQGLVPHSSLSHLAVSNSTPPPATSPVSIKVKAEPQSPPRDLSASGHQQNSNGSTGSGGSSSSTSSNASGGAGG   388
DroVir_MEF2   SYSFGAQDFSQSGAM-SADIMGLPQWHQGLVQHTSLSHLAVSNSTPPPATSPGSVKVKSEPQSPPRDLSASGHQHNSNGSTGSGSSSSSNSSNVSTTGGV   385
DroWil_MEF2   SYTFGAQDFSSSGAMSSADIMSLPQWHPGLTQHTSLSHLAVSNSTPPPATSPVSIKVKAEPQSPPRDLSASGHQHSNGSTGSGGSSSSTSSSNASASGLV   385
DroYak_MEF2   SYSFGAQDFSSSGVMNSADIMSLNTWHQGLVPHSSLSHLAVSNSTPPPATSPVSIKVKAEPQSPPRDLSASGHQQNSNGSTGSGGSSSSTSSNASGGGGA   388
DroPse_MEF2   SYSFGAQDFSSSGVIGSADIMSLPTWHQGLVQHSSLSHLAVSNSTPPPATSPVSIKVKAEPQSPPRDLSASGHQQNSNGSTGSGGSSSSTSSNASAGGGG   388
AnoG_MEF2     NYSFGAQDFS----LNPDIMIGPWSAASSHSLFSGLPHLAVSNSTPPPSTSPLSVKIKAEPISPPRDHHNTSHHHGGAPGGGSSSGASGSA---------   373
CulQ_MEF2d    NYSFGAQDFSINSDLSMIASWGAAAANNAHHSSSGLPHLAVSNSTPPPSTSPLSVKIKAEPISPPREHSSSSHHQHQHQQQQQQ---------------   368
AedA_MEF2d    NYSFGAQDFSINSDLSMIASWGAAA-SNAHHSSSGLPHLAVPNSTPPPSTSPLTVKIKAEPISPPREHQSS-----------------------------   351
TriC_MEF2     NYSFGAQDFSMGADMGLSSISWSGHQIS-HTSN-CLPHLAVSSSTPPPSSSSPIVKIKSEPISPPREHHP------------------------------   345
ApiM_MEF2     YSSFGPTDYSSDLGSLAWSHQRYVDDLSYSSSISGLPHLAVSSTPPPATSPLPVKIKSEPISPPRDPHGGNSGGSNSSGPSNTSNL--------------   365
BomM_MEF2     LTNFGPQDFSMTSDMGIGLTWGAHQLQTQHNRYIGSLPVLGGGGTPPPAASPSNVKIKAEPVSPPR-----------------------------------   332
CioI_MEF2     ----------------RNMPGMDGGNDQLSTPVVSLATPNIIQ-----QYQHAPQTSYNPVDYQVLAGGEVTGFASPGS----------------------   325
BraF_MEF2     VAIPSSRGVLPSMPQQNMRTNNQSLSTPVVSVATPSMNPGLPN-YPSAMPTAYNNDFALNSADLVGLGNFSSPISS------------------------   320
CaeB_MEF2     AYDTS------RLHPLAAADADCDLVPSSRAA-DQNIWSTNLQQRPVSQPAPSTSNLSANGISNGSSSLLSPNIS-------------------------   294
CaeE_MEF2     AYDTS------RLHPIAAADADCDLVPSSRAAADQNIWSSALQQRPVSQPAPSISNSSTNGISNGTSSLLSPNVS-------------------------   291
CaeR_MEF2     AYDTS------RLHPIAAADADCDLVPSSRAA-E-NIWSTALQQRPVSQPAPSISNSSSNGLSNGTSSLLSPNIS-------------------------   292
NasV_MEF2d    DYSTDLSSLAWSHQRYVDDLSMYSAATMSSISSL--PHLAVSSTPPPSTSPIPVKIKSEPISPPRDPHAGSGGSNGSISGPSNSSSLH-----------   331
SacK_MEF2     VTKAMTKQSPTATRPQLRANPNQSLATPTVSLATPSNPPGIPNYPSAMPTAFQSGDFALNTADLSQLSNFNSPGTSAVSA--------------------   341
GalG_MEF2     MHHL--------NTQRLGSQATHSLTTPVVSVATPSLLTQGL-PFSAMPTA-YNTDYQLTSAELSSLPAFSSPGGG------------------------   339
PhyI_MEF2     ----------------------------------------------------------YVPPSLPLYQHGMGMMGGLSGHHMLGPLPSPPNLSGILPSP   224
SchM_MEF2a    LSALSIGNQNTDNFGNRPATSAHLSPGSFSEPSSYATQRSSLTSSRRIKRPVFEPYDEYFYPKTSPNIESRSRSPILPLKQTTVSPPTSYCDSNRVTHLP   400
              .......310.......320.......330.......340.......350.......360.......370.......380.......390.......400
```

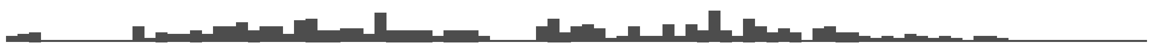

```
CanF_MEF2A     ------------------------HLGQAALSSLVAGG-----QLSQGSNLSINTNQN-INIKSEPISPPRDRMT-PSGFQQQQPQQQQ---------PP  428
MacM_MEF2A     ------------------------HLGQAALSSLVAGG-----QLSQGSNLSINTNQN-INIKSEPISPPRDRMT-PSGFQQQQQQQPP-----PPPQPQ  424
PanT_MEF2A     ------------------------HLGQAALSSLVCKS-----NLSQGSNLSINTNQN-INIKSEPISPPRDRMT-PSGFQQQQQQQPP-------PPPQ  430
SusS_MEF2A     ------------------------HLGQAALSSLVAGG-----QLSQGSNLSINTNQN-INIKSEPISPPRDRMT-PSGFQQQQPPPPS---QAPQPQPP  433
EquC_MEF2A     ------------------------HLGQAALSSLVAGG-----QLSQGSNLSINTNQN-INIKSEPISPPRDRMT-PSGFQQQQQQQQQ--------QSQ  428
PonA_MEF2A     ------------------------HLGQAALSSLVAGG-----QLSQGSNLSINTNQN-INIKSEPISPPRDRMT-PSGFQQQQQQQQQ------PPPPP  423
HomS_MEF2A     ------------------------HLGQAALSSLVAGG-----QLSQGSNLSINTNQN-ISIKSEPISPPRDRMT-PSGFQQQQQQQQQ---QQPPPPPQ  426
CalJ_MEF2A     ------------------------HLGQAALSSLVAGG-----QLSQGSNLSINTNQN-INIKSEPISPPRDRMT-PSGFQQQQQQQPP--------PPQ  421
AilM_MEF2A     ------------------------HLGQAALSSLVAGG-----QLSQGSNLSINTNQN-INIKSEPISPPRDRMT-PSGFQQQQPQP-------------  416
MusM_MEF2A     ------------------------HLGQAALSSLVAGG-----QLSQGSNLSINTNQN-INIKSEPISPPRDRMT-PSGFQQQQQQPQQ----------Q  427
DanR_MEF2A     ------------------------QLGQAALSSLVGGG-----HLPQGSNLSINTSQN-VNIKSEPISPPRERVT-PSGFPPQQQQPPS-----------  393
GalG_MEF2A     ------------------------HLGPATLSSLVSGS-----QLSQGSNLSINTNQN-INIKSEPISPPRDRVN-SSGFPQQQPPQQP----------Q  427
RatN_MEF2A     ------------------------HLGQAALSSLVAGG-----QLSQGSNLSINTNQN-INIKSEPISPPRDRMT-PSGFQQQQQQQPQ-------QQPP  421
BosT_MEF2A     ------------------------HLGQAALNSLVAGG-----QLSQGSNLSINTNQN-INIKSEPISPPRDRMT-PSGFQQQQQPQPP----------P  418
MonD_MEF2A     ------------------------HLGQAALSSLVAGG-----QLSQGSNLSINTNQN-INIKSEPISPPRDRMT-PSGFPQQQQQQQQ-----QQQQQQ  431
XenL_MEF2A     ------------------------HVGQAALSSFVATG-----QLSQGSNLSINTNQN-INIKSEPISPPRDRIT-PSGFQSHQHHQHQ-----------  416
OrnA_MEF2A     ------------------------HLGQAALGSLVAGG-----QLSQGSNLSINTNQN-INIKSEPISPPRDRMT-PSGFPQQQQQQQQ---QQQQQQQQ  434
TaeG_MEF2A     ------------------------HLGPAALSSLVTGS-----QLSQGSNLSINTNQN-INIKSEPISPPRDRVT-PSGFPPQQPQQPQP----------  427
OryC_MEF2A     ------------------------HLGQAALSSLVAGG-----QLSQGSNLSINTNQN-INIKSEPISPPRDRMT-PSGFQQQQQQQQTQPPQPQPPPPP  426
CanF_MEF2C     ------------------------HLHNMPPSALSQLGACTSTHLSQSSNLSLPSTQS-LNIKSEPVSPPRDRTTTPSR---------------------  407
MacM_MEF2C     ------------------------HLHNMPPSALSQLGACTSTHLSQSSNLSLPSTQS-LNIKSEPVSPPRDRTTTPSR---------------------  407
PanT_MEF2C     ------------------------HLHNMPPSALSQLGACTSTHLSQSSNLSLPSTQS-LNIKSEPVSPPRDRTTTPSR---------------------  405
SusS_MEF2C     ------------------------HLHNMPPSALSQLG---------------------------------DRTTTPSR---------------------  367
EquC_MEF2C     ------------------------HLHNMPPSALSQLGACTSTHLSQSSNLSLPSTQS-LNIKSEPVSPPRDRTTTPSR---------------------  407
PonA_MEF2C     ------------------------HLHNMPPSALSQLGACTSTHLSQSSNLSLPSTQS-LNIKSEPVSPPRDRTTTPSR---------------------  407
HomS_MEF2C     ------------------------HLHNMPPSALSQLGACTSTHLSQSSNLSLPSTQS-LNIKSEPVSPPRDRTTTPSR---------------------  405
CalJ_MEF2C     ------------------------HLHNMPPSALSQLGACTSTHLSQSSNLSLPSTQS-LNIKSEPVSPPRDRTTTPSR---------------------  407
AilM_MEF2C     ------------------------HLHNMPPSALSQLGACTSTHLSQSSNLSLPSTQS-LNIKSEPVSPPRDRTTTPSR---------------------  407
MusM_MEF2C     ------------------------HLHNMPPSALSQLGACTSTHLSQSSNLSLPSTQS-LSIKSEPVSPPRDRTTTPSR---------------------  399
GalG_MEF2C     ------------------------HLHNMPPSALSQLG--------------------------------------------------------------  367
RatN_MEF2C     ------------------------HLHNMPPSALSQLGACTSTHLSQSSNLSLPSTQS-LNIKSEPVSPPRDRTTTPSR---------------------  399
BosT_MEF2C     ------------------------HLHSMPPSALSQLGDRTTTPSR------------------------------------------------------  375
XenT_MEF2C     ------------------------HLHNMPHSALSQLGDRTTTPSG------------------------------------------------------  366
MonD_MEF2C     ------------------------HLHNMPPSALSQLGACTSTHLSQSTNLSLPSTQS-LNIKSEPVSPPRDRTTTPSR---------------------  407
XenL_MEF2C     ------------------------HLHNMPHSALSQLGDRTTTPSG------------------------------------------------------  363
OrnA_MEF2C     ------------------------HLHNMPPSALSQLGACTSTHVSQSTNLSLPSTQS-LHIKSEPVSPPRDRTTTPSR---------------------  407
TaeG_MEF2C     ------------------------HLHNMPPSALSQLGACTSTHLSQSTNLSLPSTQS-LNIKSEPVSPPRDRTTTPSR---------------------  407
OviA_MEF2C     ------------------------HLHNMPPSALSQLGDRTTTPSR------------------------------------------------------  367
OryC_MEF2C     ------------------------HLHNMPPSALSQLGACTSTHLSQSSNLSLPSTQS-LNIKSEPVSPPRDRTTTPSR---------------------  399
DanR_MEF2Ca    ----------------------------MQHSGLGHLGNCSSAQLCQSSALSLPSNQN-LHIKSEPVSPPRDRSGTPGL---------------------  393
DanR_MEF2Cb    ------------------------QIQNMQHSALSQLGNCSSSHLCQGSNLSLPSTQS-LHIKSEPVSPPRDRSTTPGG---------------------  385
CanF_MEF2D     -----------------------------------------------------------ACIKFEPVSPSRERSPAP-----------------------  373
MacM_MEF2D     QQQPPQPQQPQPQQPQQPQQP---PQQQSHLVPVSLSNLIPGSPLPHVGAALTVTTHPHISIKSEPVSPSRERSPAP-----------------------  453
PanT_MEF2D     QQQPPQPQQPQPQQPQQPQQP---PQQQSHLVPVSLSNLIPGSPLPHVGAALTVTTHPHISIKSEPVSPSRERSPAP-----------------------  453
SusS_MEF2D     QPQP----QP--QPQPPQQPQQPPQQQPHLVPVSLSSLIPGAPVQLGGAPLAPSGEPE-PRIAAEPSTPARERTDGH-----------------------  449
EquC_MEF2D     --------------------------------------SSPGSPLPHVGAALTVTTHPHISIKSEPVSPSRERSPAP-----------------------  395
PonA_MEF2D     QQQPPQPQQPQPQQPQQPQQP---PQQQSHLVPVSLSNLIPGSPLPHVGAALTVTTHPHISIKSEPVSPSRERSPAP-----------------------  453
HomS_MEF2D     QQQPPQPQQPQPQQPQQPQQP---PQQQSHLVPVSLSNLIPGSPLPHVGAALTVTTHPHISIKSEPVSPSRERSPAP-----------------------  453
CalJ_MEF2D     ----------------------------------------------------------------------------------------------------  371
AilM_MEF2D     ------------------------PQPQPHLVPVSLSSLIPGSPLPHVGAALTVTTHPHISIKSEPVSPSRERSPAP-----------------------  409
MusM_MEF2D     QSQPQPPQPQPQQP----------PQQQPHLVPVSLSNLIPGSPLPHVGAALTVTTHPHISIKSEPVSPSRERSPAP-----------------------  439
DanR_MEF2D     PSVSQQQTQQQQQQ----------QQQPQQLTLASLSNLVPVGTIPHGATLTVNTNPS-ISIKSEPVSPNRDRSSS------------------------  434
GalG_MEF2D     ----------------------------------------------------------------------------------------------------  352
RatN_MEF2D     QSQPQPPQPQPQQP----------PQQQPHLVPVSLSNLIPGSPLPHVGAALTVTTHPHISIKSEPVSPSRERSPAP-----------------------  439
BosT_MEF2D     QPQPPQPPQQPQQP----------PQQQPHLVPVSLSNLIPGSPLPHVGAALTITTHPHISIKSEPVSPSRERSPAP-----------------------  446
XenT_MEF2D     ------------------------HHQAQQLIPVSLSNLVSSGQLPHTATLTVNTNPN-ISIKKEPPSPNRERST-------------------------  422
MonD_MEF2D     PQQPPPQPQPQQQQQPPPPPPPP-PQQQSHLVPVSLSNLIPGSPLPHVAAALTVTTHPHISIKSEPVSPSRERSPAP-----------------------  455
TaeG_MEF2D     -----------------------------------------------------------ISIKSEPVSPNRERNTA------------------------  373
OryC_MEF2D     PQPQPPQPQPPQQPPQP-------PQQQSHLVPVSLSSLIPGSPLPHVGAALTVTTHPHISIKSEPVSPSRERSPAP-----------------------  442
XenL_MEF2Db    ------------------------HNQQQQLIPVSLSNLVSSSHLPHTATLTVNTNPN-ISIKREPASPNRERST-------------------------  422
XenL_MEF2Da    ------------------------HNQPQQLIPVSLSNLVSSSHLPHTATLTVNTNPN-ISIKREPASPNRERST-------------------------  423
CanF_MEF2B     -----------------------PPQPSGAQTLIKKGPPPRGASPRTPQLRINSKPF-------------------------------------------  292
MacM_MEF2B     -----------------------PSQPSGGRSLGEEGPPTRGASPPTPPVSIKSERL-------------------------------------------  323
PanT_MEF2B     -----------------------PSQPSGGRSLGEEGPPTRGASPPTPPVSIKSERL-------------------------------------------  323
EquC_MEF2B     -----------------------PAQPSGGRSHGREGPY-------------------------------------------------------------  305
PonA_MEF2B     -----------------------PSQPSGGRSLGEEGPPTRGASPPTPPVSIKSERL-------------------------------------------  323
HomS_MEF2B     -----------------------SSQPSGGRSLGEEGPPTRGASPPTPPVSIKSERL-------------------------------------------  323
CalJ_MEF2B     -----------------------PSQPSGGRSLGEEGPPTHSASPPTPPVSIKSERL-------------------------------------------  323
AilM_MEF2B     -----------------------PPQPSGSRSLSEEGPAARGASPPTPPVSIKSERL-------------------------------------------  320
MusM_MEF2B     -----------------------DPARPVARSLCKEGPPSRGASPPTPPVSIKSERL-------------------------------------------  304
RatN_MEF2B     -----------------------DPAGPAARSLCEEGPPSREASPPTPPVSIKSERL-------------------------------------------  304
BosT_MEF2B     -----------------------PTQPSGGRSLGEDGPPARGASSPTPPVSIKSERL-------------------------------------------  323
MonD_MEF2B     -----------------------QSHLSVGPGPGEEAPVAPGGSPPAPALSIKSERV-------------------------------------------  323
OrnA_MEF2B     -----------------------HSQLGSRIPPGDEGPPAPGVSPHPQPISIKSERV-------------------------------------------  321
DroMel_MEF2    GGAVSAANVITHLNNVSVLA-----GGPSGQGGGGGGGGSNGNVEQATNLSVLSHAQQHHLGMPNSRPSSTGHITPTPGH--------------------  463
DroSim_MEF2    GGAVSAANVITHLNNVSVLA-----GGPSGQGGGGGGGGSNGNVEQATNLSVLSHAQQHHLVMSNSRPSSTGHITPTPGH--------------------  463
DroAna_MEF2    GGGGLAAASTANVITHLNNVSVLA-GGPSGGGSGSGGGGSNGSVEQATNLSVLSHSQQQHLGMPSSRPSSTGHITPTTGH--------------------  467
DroEre_MEF2    AVGGGAVSAANVITHLNNVSVLA--GGPSGQGGGGGGGGSNGNVEQATNLSVLSHAQQHHLVMPNSRPSSTGHITPTPGH--------------------  466
DroGri_MEF2    ANVLTAINAQ---------------LGSASVLTGTPGGGGTNGSAEQATNLSVLSHSQQHLVMPNSRPSSTGHITPTPGH--------------------  448
DroMoj_MEF2    TAINAITQLGGA-------------GVLTSGGGGGGGGGGTNGSAEQATNLSVLSHSQQHLVMPNSRPSSTGHITPTPGH--------------------  451
DroPer_MEF2    AGSSAANAGNVITHLNNA-------SILGGGGPPGGGGGGSNGNAEQATNLSVLGHSQQHLVMPNSRPSSTGHLTPTPGH--------------------  461
DroSec_MEF2    GGAVSAANVITHLNNVSVLA-----GGPSGQGGGGGGGGSNGNVEQATNLSVLSHAQQHHLVMSNSRPSSTGHITPTPGH--------------------  463
DroVir_MEF2    LTAINA-------------------ITQLGGAGVGGGGGGSNGSADQATNLSVLSHSQQHLVMSNSRPSSTGHITPTPGH--------------------  446
DroWil_MEF2    TSASSVSVAANVISHLNQASV----LAGASGGGGGGGGGGSNGNAEQATNLSVLSHSQQHLVMPNSRPSSTGHITPTPGH--------------------  461
DroYak_MEF2    GGGGAVSAANVITHLNNVSVLA---GGPSGQGGGGGGGGSNGNVEQATNLSVLSHAQQHHLVMPNSRPSSTGHITPTPGH--------------------  465
DroPse_MEF2    AGSSAANAGNVITHLNNA-------SILGGGGPPGGGGGGSNGNAEQATNLSVLGHSQQHLVMPNSRPSSTGHLTPTPGH--------------------  461
AnoG_MEF2      -------------------------PGANGVQSASGGGASSTATGLTAMGNVVVTGGGGGGSGGGAGTSSLGANLNHSHLI-------------------  429
CulQ_MEF2d     -------------------------QQQQHHHHSNLSGGGGSNGQQQHNNQLTVTSMASVIGGGGHSSNSSMGNSL------------------------  419
AedA_MEF2d     -------------------------SHHHHHHSNLSGSGGGGGGGGGPGGSGGANGQQNNNQLTVTSMAQVGGHSNSQSV--------------------  406
TriC_MEF2      -------------------------HLSVGVHHSHVQSLNLTHSHHPRP---------------------------------------------------  369
ApiM_MEF2      -------------------------HHTALNVGPSSSTGAPPPHHVPHPGPQSLNLVSN-----------------------------------------  399
BomM_MEF2      --------------------------------APDHLHRGPPPASVPQPAHQMPG---------------------------------------------  355
CioI_MEF2      -------------------------LLQNWPHQSPFPQPLTTPQHLLPHPSLMTQQVPM-IRIKREPETEASPD--------------------------  373
BraF_MEF2      -----------------------WQQQHPLSAAVQAAGIPPATLSQGQSLTVQTNQGPMQIKSEPVSPPQDRHTPAV-----------------------  374
CaeB_MEF2      ----------------------------------------------------------------------------------------------------  294
CaeE_MEF2      ----------------------------------------------------------------------------------------------------  291
CaeR_MEF2      ----------------------------------------------------------------------------------------------------  292
NasV_MEF2d     -------------------------HPGHTSLNVGGPSSSSGGPQPPPPHHVSQQQQHGGQTLNLVSVSRPG----------------------------  378
SacK_MEF2      -----------------------WQHQHPLSAAVQAAGITGSTPSSSTPGLLTTDRGMGGIKSEPISPPRDKNQLNPTAGRC------------------  400
GalG_MEF2      ----------------------------------------------------------------------------------------------------  339
PhyI_MEF2      -------------TTGMLIHDFSPQNAGLFGHHPMLSGGMTGDFHDNKSSVLGLNLVPSDFNHGGAGSVHPQQLQQSKEQYYAHSKQTDSPEKSLGDRPG  311
SchM_MEF2a     LSKHLNITSWEGNQTPAIADSSRPNQSELYNNSFNLSSTSPSNRQLLSGSYDRNLSSCSNLKCKDDPYVTLIRDTQNSSHNLQTSSSTPSMQERFSHFPV  500
               .......410.......420.......430.......440.......450.......460.......470.......480.......490.......500
```

```
CanF_MEF2A   PPQPSQPPQPRQEMGRSPVDSLSSSSS-SYDGSDREDPRGDFHSPIVLGRPPNTEDRESPSVKRMRM-DAWVT   499
MacM_MEF2A   PQPQPPQPQPRQEMGRSPVDSLSSSSS-SYDGSDREDPRGDFHSPIVLGRPPNTEDRESPSVKRMRM-DAWVT   495
PanT_MEF2A   PQPQPPQPQPRQEMGRSPVDSLSSSSS-SYDGSDREDPRGDFHSPIVLGRPPNTEDRESPSVKRMRM-DAWVT   501
SusS_MEF2A   QPQPQPQPQARQEMGRSPVDSLSSSSS-SYDGSDREDPRGDFHSPVVLGRPPNTEDRESPSVKRMRM-DAWVT   504
EquC_MEF2A   PPQPQPQPQPRQEMGRSPVDSLSSSSS-SYDGSDREDPRGDFHSPIVLGRPPNTEDRESPSVKRMRM-DAWVT   499
PonA_MEF2A   PQPQPPQPQPRQEMGRSPVDSLSSSSS-SYDGSDREDPRGDFHSPIVLGRPPNTEDRESPSVKRMRM-DAWVT   494
HomS_MEF2A   PQPQPPQPQPRQEMGRSPVDSLSSSSS-SYDGSDREDPRGDFHSPIVLGRPPNTEDRESPSVKRMRM-DAWVT   497
CalJ_MEF2A   PQPQPQQPQPRQEMGRSPVDSLSSSSS-SYDGSDREDPRGDFHSPIVLGRPPNAEDRESPSVKRMRM-DAWVT   492
AilM_MEF2A   -------------------------------------------------------------------------   416
MusM_MEF2A   PPPQPPQPQPRQEMGRSPVDSLSSSSS-SYDGSDREDPRGDFHSPIVLGRPPNTEDRESPSVKRMRM-DTWVT   498
DanR_MEF2A   ---------GRPDMGRSPVDSLSSSCS-SYDGSDREDHRPDFHSPLGLGRPPAADERESPSVKRMRM-DTWVT   455
GalG_MEF2A   PPQPPQQPPQRQEMGRSPVDSLSSSSS-SYDGSDREDPRSDFHSPVVLGRPPNSEDRESPSVKRMRM-DTWVT   498
RatN_MEF2A   PQPPQPQPQPRQEMGRSPVDSLSSSSS-SYDGSDREDPRGDFHSPIVLGRPPNAEDRESPSVKRMRM-DTWVT   492
BosT_MEF2A   PPPQAPQPQPRQEVGRSPVDSLSSSSS-SYDGSDREDPRGDFHSPVVLGRPPNSEDRESPSVKRMRM-DAWVT   489
MonD_MEF2A   QQQQQQQQQARQEMGRSPVDSLSSSSS-SYDGSDREDPRSDFHSPIGLGRPPNTEDRESPSVKRMRM-DTWVT   502
XenL_MEF2A   ---------PRPEM-----DSLSSSSS-SYDGSDREDVRNDFHSPIGLGRPANNEDRDSPSVKRMRM-DAWVT   473
OrnA_MEF2A   QQQPQQQQSSRQEIGRSPVDSLSSSSS-SYDGSDREDPRSDFHSPIGLGRPPNTEDRESPSVKRMRM-DTWVT   505
TaeG_MEF2A   -----PPPPSRQELGRSPVDSLSSSSS-SYDGSDREDPRSDFHSPVVLGRPPNAEDRESPSVKRMRM-DTWVT   493
OryC_MEF2A   PQPPQPQPQPRQDMGRSPVDSLSSSSS-SYDGSDREDPRGDFHSPIVLGRPPNPEDRESPSVKRMRM-DAWVT   497
CanF_MEF2C   -----YPQHTRHEAGRSPVDSLSSCSS-SYDGSDREDHRNEFHSPIGLTRP-SPDERESPSVKRMRLSEGWAT   473
MacM_MEF2C   -----YPQHTRHEAGRSPVDSLSSCSS-SYDGSDREDHRNEFHSPIGLTRP-SPDERESPSVKRMRLSEGWAT   473
PanT_MEF2C   -----YPQHTRHEAGRSPVDSLSSCSS-SYDGSDREDHRNEFHSPIGLTRP-SPDERESPSVKRMRLSEGWAT   471
SusS_MEF2C   -----YPQHTRHEAGRSPVDSLSSCSS-SYDGSDREDHRNEFHSPIGLTRP-SPDERESPSVKRMRLSEGWAT   433
EquC_MEF2C   -----YPQHTRHEAGRSPVDSLSSCSS-SYDGSDREDHRNEFHSPIGLTRP-SPDERESPSVKRMRLSEGWAT   473
PonA_MEF2C   -----YPQHTRHEAGRSPVDSLSSCSS-SYDGSDREDHRNEFHSPIGLTRP-SPDERESPSVKRMRLSEGWAT   473
HomS_MEF2C   -----YPQHTRHEAGRSPVDSLSSCSS-SYDGSDREDHRNEFHSPIGLTRP-SPDERESPSVKRMRLSEGWAT   471
CalJ_MEF2C   -----YPQHTRHEAGRSPVDSLSSCSS-SYDGSDREDHRNEFHSPIGLTRP-SPDERESPSVKRMRLSEGWAT   473
AilM_MEF2C   -----YPQHTRHEAGRSPVDSLSSCSS-SYDGSDREDHRNEFHSPIGLTRP-SPDERESPSVKRMRLSEGWAT   473
MusM_MEF2C   -----YPQHTRHEAGRSPVDSLSSCSS-SYDGSDREDHRNEFHSPIGLTRP-SPDERESPSVKRMRLSEGWAT   465
GalG_MEF2C   -------------------------------------------------------------------------   367
RatN_MEF2C   -----YPQHTRHEAGRSPVDSLSSCSS-SYDGSDREDHRNEFHSPIGLTRP-SPDERESPSVKRMRLSEGWAT   465
BosT_MEF2C   -----YPQHTRHEAGRSPVDSLSSCSS-SYDGSDREDHRNEFHSPIGLTRP-SPDERESPSVKRMRLSEGWAT   441
XenT_MEF2C   -----YPQHTRHEAGRSPVDSLSSCSS-SYDGSDREDHRNDFHSPIGLTRP-SPDERESPSVKRMRLSEGWAT   432
MonD_MEF2C   -----YPQHTRHEAGRSPVDSLSSCSS-SYDGSDREDHRNEFHSPIGLTRP-SPDERESPSVKRMRLSEGWAT   473
XenL_MEF2C   -----YPHHTRHDAGRSPVDSLSSCSS-SYDGSDREDHRNDFHSPIGLTRP-LQDERESPSVKRMRLSEGWAT   429
OrnA_MEF2C   -----YPQHTRHEAGRSPVDSLSSCSS-SYDGSDREDHRNEFHSPIGLTRP-SPDERESPSVKRMRLSEGWAT   473
TaeG_MEF2C   -----YQQHTRHEAGRSPVDSLSSCSS-SYDGSDREDHRNEFHSPIGLTRP-SPDERESPSVKRMRLSEGWAT   473
OviA_MEF2C   -----YPQHTRHEAGRSPVDSLSSCSS-SYDGSDREDHRNEFHSPIGLTRP-SPDERESPSVKRMRLSEGWAT   433
OryC_MEF2C   -----YPPHTRHEAGRSPVDSLSSCSS-SYDGSDREDHRNEFHSPIGLTRP-SPDERESPSVKRMRLSEGWAT   465
DanR_MEF2Ca  -----YPQQTRQDSGRSPVDSLSSCGS-SYDGSDRDEHRANFHSPVGLMRAAEN-ERQSPSVKRMRLSEGWAS   459
DanR_MEF2Cb  -----YPSHQRQDSGRSPADSLSSCGS-SHEGSDRDEHRPDFHSPLGLGRPGLD-DQDSPSIKRVRLSEGWAT   451
CanF_MEF2D   -----PPPAVFPATRPEPGDGLSSPASSSYETGDRDDGRGDFGPTLGLLRPAPEPEAEGPAVKRM-RLDTWDA   440
MacM_MEF2D   -----PPPAVFPAARPEPGDGLSSPAGGSYETGDRDDGRGDFGPTLGLLRPAPEPEAEGSAVKRM-RLDTWVL   520
PanT_MEF2D   -----PPPAVFPAARPEPGDGLSSPAGGSYETGDRDDGRGDFGPTLGLLRPAPEPEAEGSAVKRM-RLDTWTL   520
SusS_MEF2D   -----PTRSLFQAARIQPGAGLSTPAQASAAAAERLHGRGKAGRTLGLQGPEEDPAAVGPQDRTV-GATFHFL   516
EquC_MEF2D   -----PPPAVFPATRPEPGDGLSSPAGGSYETGDRDDGRGDFGPTLGLLRPAPEPEAEGSAVKRM-RLDTWGL   462
PonA_MEF2D   -----PPPAVFPAARPEPGDGLSSPAGGSYETGDRDDGRGDFGPTLGLLRPAPEPEAEGSAVKRM-RLDTWVL   520
HomS_MEF2D   -----PPPAVFPAARPEPGDGLSSPAGGSYETGDRDDGRGDFGPTLGLLRPAPEPEAEGSAVKRM-RLDTWTL   520
CalJ_MEF2D   ----------------------------QPQQPQPPQQQPPQPQQPQPQQPQQPQQPPQPQSHLVPVSLSNLM   416
AilM_MEF2D   -----PPPAVFPAARPEPGDGLSSPASGSYETGDRDDGRGDFGPTLGLLRPAQEPEAEGPAVKRMRCSNGAG-   476
MusM_MEF2D   -----PPPAVFPAARPEPGEGLSSPAGGSYETGDRDDGRGDFGPTLGLLRPAPEPEAEGSAVKRM-RLDTWTL   506
DanR_MEF2D   -----TPTVSQYPTLRLEPGRSISSNGSSYEGSDRDDSGQPDFSSLGLLRATGDQQQESANVKRM-RLDTWVT   501
GalG_MEF2D   -------------------------------------------------------------------------   352
RatN_MEF2D   -----PPPAVFPAARPEPGEGLSSPAGGSYETGDRDDGRGDFGPTLGLLRPAPEPEAEGSAVKRM-RLDTWTL   506
BosT_MEF2D   -----PPPAVFPATRPEPGDALSSPAGGSYETGDRDDGRGDFGPTLGLLRPAPEPEAEGSAVKRM-RLDTWTL   513
XenT_MEF2D   -----GTPLSSFPQSRHEVGRSLSSNASSFEGNDREDVRGDYTSSLGLLRPSGDTESESQSVKRM-RLDAWVT   489
MonD_MEF2D   -----PAPAPFPPARPEPGDGLSSPASASYEAGERDDGRGDFGPTLGLLRPAPEPDTESSAVKRM-RLDTWVT   522
TaeG_MEF2D   -----TPLSTFPHQPRHEPGRSLSSNTSSYEGSERDDPARDFGSSLGLLRPTSEPEGESPSVKRM-RLDTWVT   440
OryC_MEF2D   -----PPPAVFPAARPEPDDSLSSPAGGAYETGDRDDGRGDFGPTLGLLRPAPEPDAEGSAVKRM-RLDTWVL   509
XenL_MEF2Db  -----GTPLSCFPQSRHEVGRSLSSNASSFEGNDREDPRGDYTSSLGLLRPSGDTESESQSVKRM-RLDAWVT   489
XenL_MEF2Da  -----GTPLSCFSQSRHEAGRSLSSNASSFEGNDREDPRGDYTSSLGLLRPSGDTESESQSVKRM-RLDAWVT   490
CanF_MEF2B   ---------------------------SPPPGAPENFPKIFPYPLFFSRPLGKPLRPGPPLPEFPIGNAWPR-   337
MacM_MEF2B   ---------------------------SPAPGGPGDFPKTFPYPLLLARSLAEPLRPGPALRRLPLADGWPR-   368
PanT_MEF2B   ---------------------------SPAPGGPGDFPKTFPYPLLLARSLAEPLRPGPALRRLPLADGWPR-   368
EquC_MEF2B   -------------------------------------------------------------------------   305
PonA_MEF2B   ---------------------------SPAPGGPGDFPKTFPYPLLLARSLAEPLRPGPVLRRLPLADGWPR-   368
HomS_MEF2B   ---------------------------SPAPGGPGDFPKTFPYPLLLARSLAEPLRPGPALRRLPLADGWPR-   368
CalJ_MEF2B   ---------------------------SPAPGGPGDFPKTFPYPLLLARSLAEPLRPGPALRRLPLADGWPR-   368
AilM_MEF2B   ---------------------------SPAPGGPGDFPKTFPYPLLLSRPLAEPLRPGPPLRRLPTADGWAR-   365
MusM_MEF2B   ---------------------------SPVTGTSGDFPRSFPYPLLLARPLAEPLRPSASLHRL-TPDSWPR-   348
RatN_MEF2B   ---------------------------SPVTGTSGDFPRSFPYPLLLARPLAEPLRP-ASLRRL-TPDSWPR-   347
BosT_MEF2B   ---------------------------SPAPGGPGDFPKTFPYPLLLARPLAEPLRPGPPLRRLPTADGWPR-   368
MonD_MEF2B   ---------------------------SPGAVPRDDFTKSYQYPVGLARP-------GQPLRRLHVADSWPR-   361
OrnA_MEF2B   ---------------------------SPGATCREDFTKGYPYPLVLARPLNEEPRTGLPTRRLQVAEMVVV-   366
DroMel_MEF2  ------DKYEGYPYRALMGHNPRWNFAGAPSSDQDVRLAAVAQQQQQQPHQQQQLGDYDAPNHKRPRISGGWG   530
DroSim_MEF2  ------DKYEGYPYRALMGHNPRWNFAGAPSSDQDVRLAAVAQQQQQQQHQQQQLGDYDAPNHKRPRISGGWG   530
DroAna_MEF2  ------DKYDGYPYRALMGHNPRWNFAGAPSSDQDVRMAAAAHQQQQQQQQQQQLGDYDAPNHKRPRISGGFG   534
DroEre_MEF2  ------DKYEGYPYRALMGHNPRWNFAGAPSSDQDVRLAAVAQQQQQQQHQQQQLGDYDAPNHKRPRISGGWG   533
DroGri_MEF2  ------DKYEGYPYRALMGHNSRWNFAGAPGSEQDVRLAAVAQQQHQQQQQQQQLSDYDAPNHKRPRISGGWG   515
DroMoj_MEF2  ------DKYEGYPYRALMGHNPRWNFAGAPNGEQDVRLQAVAQQQQQQHQQQQQLSDYDAPNHKRPRISGGWG   518
DroPer_MEF2  ------DKYEGYQYRLLMGHNPRWNSAGAPSSEQDVRLAAVAQQHQQHQQQQQQLGDYDAPNHKRPRISGGWG   528
DroSec_MEF2  ------DKYEGYPYRALMGHNPRWNFAGAPSSDQDVRLAAVAQQQQQQQHQQQQLGDYDAPNHKRPRISGGWG   530
DroVir_MEF2  ------DKYEGYQYRALMGHNPRWNFAGAPSSEQDVRLAAVAQQQQQHQQQQQQLSDYDAPNHKRPRISGGWG   513
DroWil_MEF2  ------DKYEGYPYRALMGHNPRWNFAGTPTSEQDVRLQQQHQQQQQHQQQQQLNDYDAPHQKRPRISGGWA   528
DroYak_MEF2  ------DKYEGYPYRALMGHNPRWNFAGAPSSDQDVRLAAVAQQQQQQQHQQQQLGDYDAPNHKRPRISGGWG   532
DroPse_MEF2  ------DKYEGYQYRLLMGHNPRWNSAGAPSSEQDVRLAAVA-QHQQHQQQQQQLGDYDAPNHKRPRISGGWG   527
AnoG_MEF2    ------HSRPSSTGHLTPTPDSYMPIGGHLAGSVTPTNAPSPVDIRHLSTGGSHLPEYDAPTHKRPRISEGWA   496
CulQ_MEF2d   ------NHSHLIHSRPSSTGHLTPTPGSVTPTNAPSPVDIRHINVGGGGGNSSHLPDYDSPVHKRPRISEGWA   486
AedA_MEF2d   ------ALGSSLNHSHLIHSRPSSTGHLTPTPGSVTPTNAPSPVDIRHLGGGGPLPDYDSPVHKRPRISEGWA   473
TriC_MEF2    ----------------------------SSAGHITPTPGSVTPTNLPSPTGPPSGGDYENIQHKRARLSDWP   413
ApiM_MEF2    -----------RPSSNPPPSHSGSITPTNLPSPGSATVADIRTSHSNTGGNGGNNSDYENGPMKRSRITEGWA   461
BomM_MEF2    ----------------------------VIDGSVSSSNMGSPAGQDMRHSNAVALDYEQPHTKRP-RIEGWA   398
CioI_MEF2    ------KDPDQRPAAGRSPMEVMSPYEMRMDDRSMMVENGGNLPVKRQRVESGPN-----------ASPTWT   428
BraF_MEF2    ----NNQGGLHANNTSPVNSLCSSSSSSPFPADGEDRGDGSFHSPGGSRMRKAMEDEGRGMRLSL--------   435
CaeB_MEF2    ---------------------------SLNGHSVLDLGGPIPPYKMDPNAYIKVEPHSPPQKRPRITTEWRQQ   340
CaeE_MEF2    ---------------------------SLNGHSVLDLGGPNLPYKLDPNTYVKMEPHSPPEKRPRITTEWRPQ   337
CaeR_MEF2    ---------------------------SLNGHSVLDLGGPIPPYKLDPNAYVKLEPHSPPQKRPRITTEWRPQ   338
NasV_MEF2d   ------SNPPPSHSGSITPTNLPSPGPQGGGGGGNGGVVVGDRTSHGPGASGGSGSDYENGPHKRSRITEGWA   445
SacK_MEF2    ----TPHDTAMSPGRHSHCSSASASPFAGSDREDGRGGGDSNYQSSGNRGSIMGDKDGPPTKRQRIENAWST-   468
GalG_MEF2    ---------------------------NISAWQQQQQQQQQQQQQQQQQQQQQQQQQQQQQQ---------   376
PhyI_MEF2    SPEASGDKPSDEVVCKVEKPTEKEQSRQDDESNNEDSQSAKENETTDKAKSLSPKRE---------------   368
SchM_MEF2a   IHESIASLNSCKIQQQPPQQLQNPPNILDDEIGVGSIPSNQRSFKPDQFIESVPSPELLLFLSSNNNNSNNLK   573
             .......510.......520.......530.......540.......550.......560.......570...
```

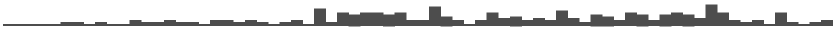

Supplement: File S2 — Alignment result for the 102 MEF2 protein sequences. (PDF) [file pone.0017334.s002.pdf]
