# Supplementary figures and images for: Vertebrate Paralogous MEF2 Genes: Origin, Conservation, and Evolution
Source: PLoS One. 2011 Mar 4;6(3):e17334. doi: 10.1371/journal.pone.0017334 (PMC3048864; doi:10.1371/journal.pone.0017334)

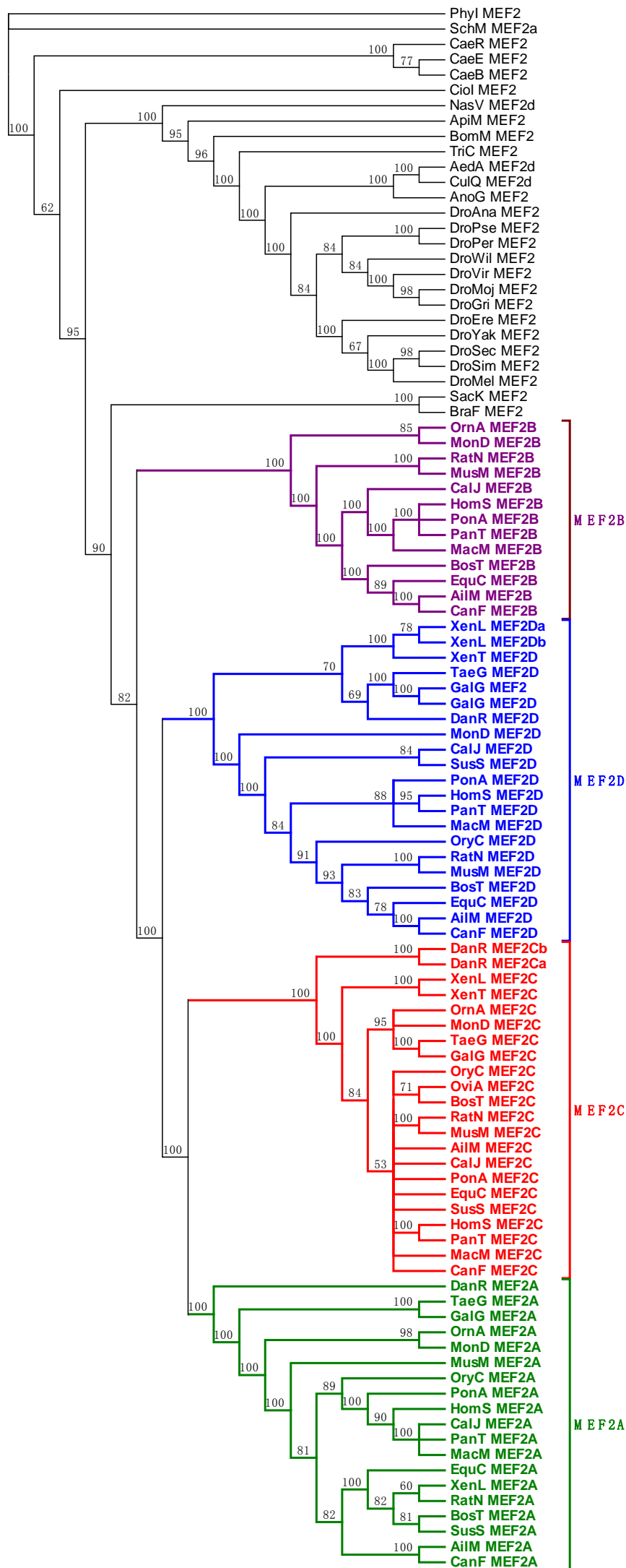

Supplement: Figure S1 — MEF2 Bayesian tree. (PDF) [file pone.0017334.s005.pdf]

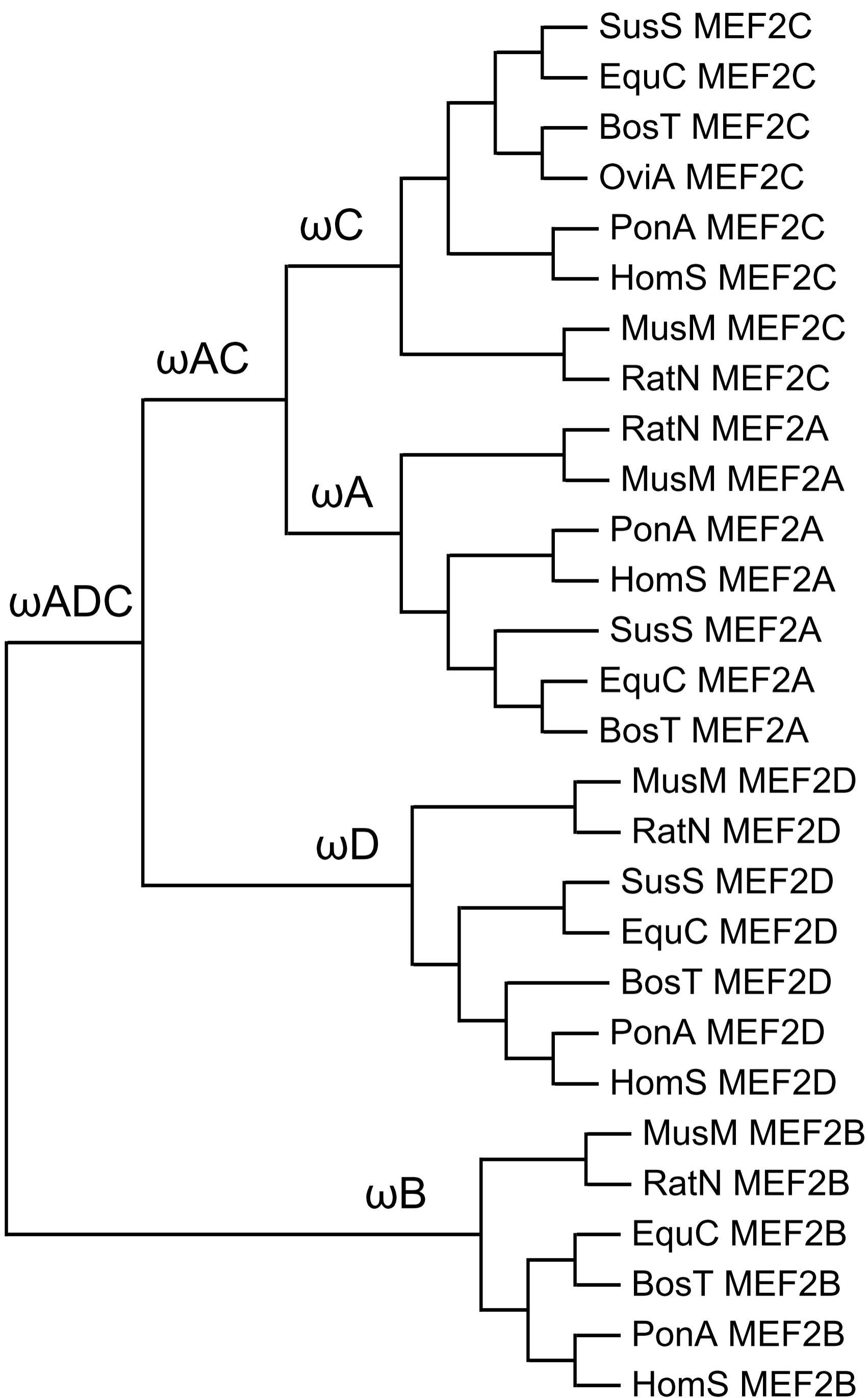

Supplement: Figure S2 — Different ω parameters for different parts of the MEF2A-D phylogeny. (PDF) [file pone.0017334.s006.pdf]
