# Supplementary material for: Vertebrate Paralogous MEF2 Genes: Origin, Conservation, and Evolution
Source: PLoS One. 2011 Mar 4;6(3):e17334. doi: 10.1371/journal.pone.0017334 (PMC3048864; doi:10.1371/journal.pone.0017334)
Supplement: File S4 — Alignment result of the MADS and MEF2s coding regions for the MEF2A, B, C, and D branches. (PDF) [file pone.0017334.s004.pdf]

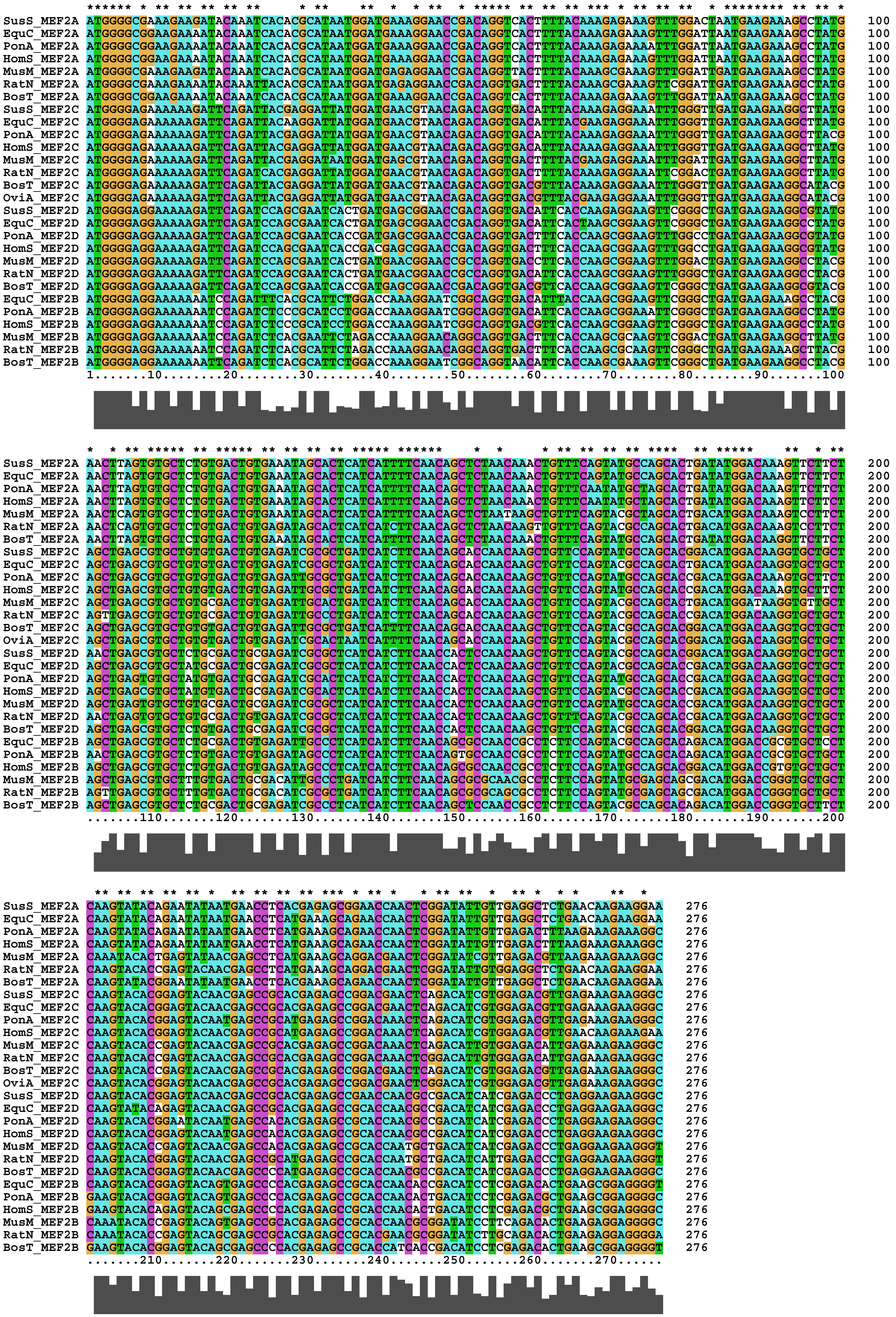
SusS_MEF2A
EquC_MEF2A
PonA_MEF2A
HomS_MEF2A
MusM_MEF2A
RatN_MEF2A
BosT_MEF2A
SusS_MEF2C
EquC_MEF2C
PonA_MEF2C
HomS_MEF2C
MusM_MEF2C
RatN_MEF2C
BosT_MEF2C
OviA_MEF2C
SusS_MEF2D
EquC_MEF2D
PonA_MEF2D
HomS_MEF2D
MusM_MEF2D
RatN_MEF2D
BosT_MEF2D
EquC_MEF2B
PonA_MEF2B
HomS_MEF2B
MusM_MEF2B
RatN_MEF2B
BosT_MEF2B
100
200
276
